# Urban Trees and Human Health: A Scoping Review

**DOI:** 10.3390/ijerph17124371

**Published:** 2020-06-18

**Authors:** Kathleen L. Wolf, Sharon T. Lam, Jennifer K. McKeen, Gregory R.A. Richardson, Matilda van den Bosch, Adrina C. Bardekjian

**Affiliations:** 1 School of Environmental and Forest Sciences, College of the Environment, University of Washington, Seattle, WA 98195, USA; 2 Ontario Climate Consortium Secretariat, Toronto and Region Conservation Authority, Toronto, ON L4K 5R6, Canada; sharon.lam@mail.utoronto.ca; 3 Canadian Forest Service, Natural Resources Canada (Government of Canada), Vancouver, BC V6B 5J3, Canada; jmckeen@ualberta.ca; 4 Climate Change and Innovation Bureau, Health Canada (Government of Canada), Ottawa, ON K1Y 4X2, Canada; gregory.richardson@canada.ca; 5 School of Population and Public Health, The University of British Columbia, Vancouver, BC V6T 1Z3, Canada; matilda.vandenbosch@ubc.ca; 6 Department of Forest and Conservation Sciences, The University of British Columbia, Vancouver, BC V6T 1Z4, Canada; 7 Engagement and Research, Tree Canada, Ottawa, ON K1R 6S3, Canada; abardekjian@treecanada.ca

**Keywords:** urban forest, green infrastructure, urban greening, greenspace, ecosystem services, public health, social determinant, health promotion

## Abstract

The urban forest is a green infrastructure system that delivers multiple environmental, economic, social and health services, and functions in cities. Environmental benefits of urban trees are well understood, but no review to date has examined how urban trees affect human health. This review provides a comprehensive summary of existing literature on the health impacts of urban trees that can inform future research, policy, and nature-based public health interventions. A systematic search used keywords representing human health, environmental health, and urban forestry. Following screening and appraisal of several thousand articles, 201 studies were conceptually sorted into a three-part framework. Reducing Harm, representing 41% of studies, includes topics such as air pollution, ultraviolet radiation, heat exposure, and pollen. Restoring Capacities, at 31%, includes attention restoration, mental health, stress reduction, and clinical outcomes. Building Capacities, at 28%, includes topics such as birth outcomes, active living, and weight status. The studies that were reviewed show substantial heterogeneity in purpose and method yet indicate important health outcomes associated with people’s exposure to trees. This review will help inform future research and practice, and demonstrates why urban forest planning and management should strategically promote trees as a social determinant of public health.

## 1. Introduction

Trees are some of the most prominent natural features in towns and cities from both visual and functional perspectives. The urban forest is a key type of green infrastructure system [1] and a consequential component of other urban ecosystems and landscapes [2,3,4]. Comprised of diverse tree species and vegetation structures, the urban forest includes individual trees, assemblages of trees in parks, groves, and extensively forested natural areas, which are distributed across public and private properties and along streets, waterfronts, railways, and riverbanks [5,6]. 

The ecological functions and services of urban forests have been investigated extensively in recent decades [7,8,9,10,11]. Benefits include the ability of trees to reduce greenhouse gases through carbon storage [12,13,14], decrease stormwater runoff through interception and absorption of rainwater [15], and mitigate the urban heat island effect through reductions in surface and air temperatures at a local scale [16,17]. However, knowledge about the relationship between urban trees and human health is still developing. The academic literature on the linkages between nature and human health has grown rapidly using various specifications of nature, such as urban greening, green space, open space, parks, therapeutic landscapes, and restorative settings. As the evidence base has expanded, reviews have consolidated knowledge of associated health outcomes, but many have focused broadly on nature [17,18,19,20,21,22], green space [23,24,25,26,27,28], and greenness [22,29].

More information about specific qualities of urban tree conditions and exposures are needed in order to help guide and inform planning, design, and implementation decisions. Local governments and other organizations show increased interest in promoting and enhancing community-based nature as a social determinant of health [30,31]. From a practical standpoint, effective implementation requires better articulation of specific elements of nature and how they may influence health outcomes. Policy, professional staffing, and budgets are often allocated less to generalities of nature and more specifically to departmental administrations addressing parks, trees, vegetation in rights-of-way, natural areas or landscapes associated with development.

While other studies have focused on nature, green space and greenness, no comprehensive review has assessed the full range of evidence on the human health responses associated specifically with trees in urban areas. A review by Salmond et al. focused on street trees and health [32] but did not address other forms and configurations of the urban forest. To address this gap in the literature, this scoping review synthesizes empirical findings about how urban trees and forest experiences can impact human health. Scoping reviews are used to assess “the extent, range, and nature of research activity in a topic area” [33] (p. 371) and are used for ‘reconnaissance’ to clarify working definitions and conceptual boundaries of a topic or field, especially for a large and diverse body of literature that is not amenable to a systematic review [34,35]. We conducted a literature screening across varied disciplines (including epidemiology, medicine, environmental and atmospheric sciences, psychology, and other social sciences), sources and study methods, then summarized results in a conceptual framework that can inform future research questions and methods.

This scoping review also has practical implications. In the United States, urban tree canopy cover is estimated to be declining at a rate of roughly four million trees per year due to rapid urbanization and tree diseases and pests [36]; other countries may be experiencing similar losses. Understanding how urban trees are associated with human health can inform how health professionals, urban foresters, town and city planners, and urban designers can maximize the public health benefits generated by urban trees by supporting better tree policy, planning, and management.

## 2. Methods 

### 2.1. Search and Inclusion Strategies

The review was conducted in accordance with PRISMA-ScR (Preferred Reporting Items for Systematic Reviews and Meta-Analyses) [37]. Published findings of quantified relationships between urban trees and human health were collected by searching Embase, Ovid Medline, PsycINFO, and PubMed. Keywords used included terms related to trees, land cover, urban settings, public health and known health outcomes (Table 1). 

**Figure 1:**
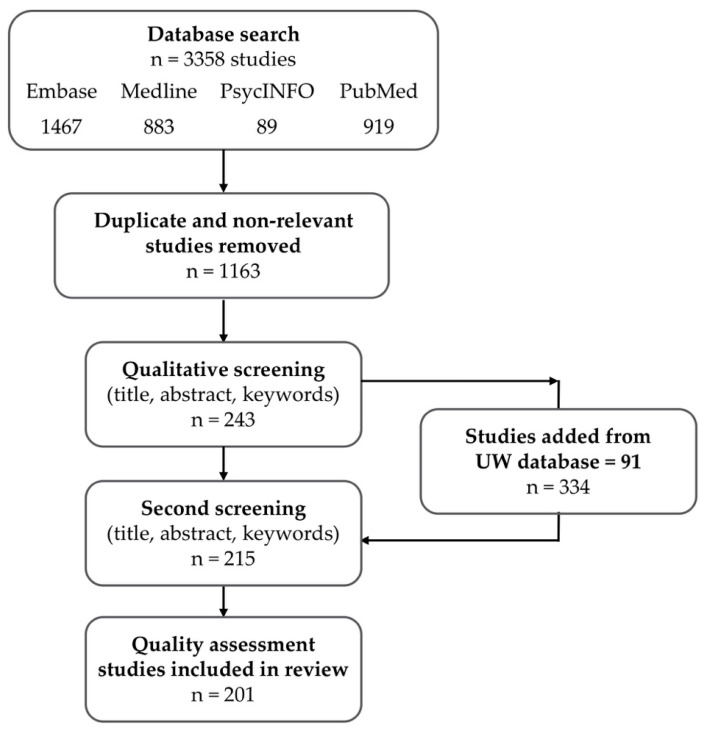
Search and inclusion process for a scoping review of urban trees and human health.

The initial search produced a collection of 3358 peer-reviewed articles; the number was reduced to 1663 after duplicates and non-relevant publications were removed (Figure 1). The resulting article collection was limited to English language and peer-reviewed journal articles; no other limits were applied. 

Search results were qualitatively screened independently by two authors based on titles, abstracts, keywords and study approaches. Articles were retained based on the following inclusion criteria: (a) original research article; (b) use of quantitative or mixed-methods to quantify the associations between urban trees and human health; (c) with “human health” referring broadly to illness, disease, symptoms, and wellness pathways such as improved physical and mental health conditions, healthy behaviors, and known environmental health risks (e.g., air quality, heat, ultraviolet radiation (UVR) exposure, and noise); (d) tree exposure measures (i.e., subjective and objective) or an explicit reference to trees in the findings; (e) with “trees” expressed as streetscapes, single trees, canopy cover, trees in a park, forested natural areas, and photos or videos of trees and landscapes. Note that studies using measures of vegetation cover (such as normalized difference vegetation index (NDVI)) were only included if results specified tree-associated outcomes; (f) with “urban” expressed as a study conducted in urban settings and/or comprised of urban populations; and g) all potential human beneficiary populations (i.e., age, gender, cultural background, and socio-economic status). 

Studies that implied health outcomes but did not specifically include evaluation of health consequences were excluded. For example, studies on the relationship between urban trees and air quality that measured or modeled air pollution levels but did not include measures of human response or explicitly call out the health implications of the pollution levels, were excluded. After all articles were independently screened, the lists were compared and disagreements about inclusion or exclusion were resolved through discussion and consensus by three authors. The qualitative screening reduced the article count to 243. As expected for scoping reviews, we conducted multiple structured searches for relevant literature [35]. The screening revealed notable omission of articles from the environmental sciences. The database that supports the Green Cities: Good Health web site at the University of Washington was subsequently searched, adding another 91 articles. After again screening for duplicates and content relevance, the collection totaled 215 articles, all published prior to 1 March, 2018.

### 2.2. Quality Assessment

We then conducted a science quality assessment using a modified version of the Effective Public Health Practice Project Quality Assessment Tool [38], a public health research assessment tool for quantitative studies. Collected articles originated from multiple disciplines, including public health, social sciences, neuroscience, and environmental sciences, and encompassed many types of study designs, ranging from experimental to cross-sectional studies. We therefore modified the tool with the aim of broadening its applicability and focused on five components: (a) study design, (b) sample selection, (c) confounds, (d) data collection method, and (e) analyses. Each paper received a score of either: 1 (strong), 2 (moderate), or 3 (weak) for each component, and a global rating: strong (no weak ratings), moderate (one weak rating), or weak (two or more weak ratings).

The quality assessment was conducted independently by two authors and disagreements about scores were resolved through discussion and consensus. Inter-rater reliability for the global ratings was assessed using Cohen’s Kappa and was found to be satisfactory (κ = 0.929, *p* < 0.0001). Only studies that received a strong global rating (i.e., no weak component ratings) were retained. Sixteen articles were excluded, leaving 199 articles—for a total of 201 studies (as two papers reported multiple studies)—for subsequent analysis.

### 2.3. Thematic Analysis

To synthesize the resulting diverse literature into a coherent narrative, we adopted the conceptual framework published by Markevych et al. [39], after considering multiple existing nature and health frameworks. The intentionally comprehensive framework by Markevych et al. was developed during a transdisciplinary expert workshop, with a particular focus on potential underlying biopsychosocial pathways linking green space to health. The domains reflect, but also expand on, prior empirical foundations. Each of the three domains imply ways to modify environments to support adaptation and promote health. The first, Reducing Harm, considers the role of vegetation in mitigating the conditions that can compromise health, and includes concerns of exposure to air pollution, noise, and heat. The second, Restoring Capacities, describes how nature experiences are a resource that promotes improved psychological and physiological functioning, including cognitive attention restoration, and stress recovery. The last domain, Building Capacities, describes nature experience pathways that facilitate multiple conditions of wellness for both individuals and communities, such as encouraging physical activity and providing settings for social cohesion. Sorting of articles into these domains was conducted by one author and confirmed by two others.

## 3. Results—Study Attributes

### 3.1. Study Locations

Of the 201 studies, 39% were based in North America, with 67 studies undertaken in the United States, 9 in Canada, and 1 in Mexico. About one third were conducted in Asia, including Japan (17% of the entire collection), South Korea (7%), China (4%), and Taiwan (3%). Another 25 %of studies were based in Europe including Spain, the United Kingdom, Sweden, Germany, Portugal, and France. The remaining studies were based in Australia (3%), and South America (1%). No study was based in Africa, and one study did not specify a location.

### 3.2. Study Participants

The full range of the human life span was represented, as 13% of studies focused on young adults and 13% on children. Adults were the primary age group studied (71% of studies), with 3% focusing on older adults. Studies of all-male participants (8%) were more frequent than all-female (3%). Forest bathing experiments were prominent among studies based in Asia, and commonly included young adult-male samples. Forty percent of studies involved participants with pre-existing medical conditions. Controlling for socio-economic factors was common among cross-sectional studies. However, few studies included a detailed analysis of how urban trees impact specific sub-populations (e.g., race/ethnicity, socio-economic status, or other factors of health risk).

### 3.3. Outcomes Measures

Various human response measures were used in the studies (with some designs using more than one): (a) psychological tests, including questionnaires and cognitive assessments of stress, job satisfaction, and mental acuity (29%); (b) physiological measures such as heart rate, cortisol, adrenalin, and glucose levels (27%); (c) self-reported symptoms of illness and allergies (14%); (d) modelling of human health impacts related to heat and air quality (each 5%) using primary and secondary data; (e) actual air quality using primary and secondary data (45%); (f) hospitalization and medical records (7%); (g) medication usage based on drug prescription/sales data (3%); and (h) neurological measures such as functional Magnetic Resonance Imaging (fMRI) scans (3%). Additional measures, each representing less than 2% of the collection were: (i) syndromic surveillance records such as ambulance calls and emergency room visits; (j) therapy effectiveness, such as Cognitive Behavioral Therapy; and (k) measures of participant-experienced ultraviolet radiation.

### 3.4. Tree Exposures

The 201 studies characterized exposure to urban trees and forests in various ways, with some studies using multiple independent variables. Tree exposure was measured both objectively (e.g., using satellite imagery, other remote sensing, and geographic information systems), and subjectively (e.g., surveys). Tree presence was also characterized based on quantity (e.g., density, proximity), as well as quality (e.g., well/not maintained). Specific tree measures included: (a) experience or a visit within a forest or woodland (e.g., walking, climbing, social activity, therapy) (27%); (b) canopy cover (16%); (c) individual/clusters of trees (e.g., street trees, schoolyard trees) (14%); (d) associated measures such as pollen, moss, and tree loss to emerald ash borer (EAB) (18%); (e) viewing images/simulations of trees/landscapes (8%); (f) forest/woodland/land cover using satellite sensors (such as NDVI), LiDAR and other remote sensing technologies (7%); (g) experiencing trees in a park, open space or natural area (4%); and (h) view of trees/forest through a window (1%).

### 3.5. Study Designs

A variety of study designs were observed (Table 2). Experimental studies (28% of the study collection) used both objective and self-report measures, and involved forest visits, tree views, or viewing simulations (e.g., videos or images of trees) as response stimulus or interventions. Sample sizes ranged from 8 to 625 participants. Within subjects, crossover studies of forest versus built environments were the most commonly used design among experimental studies. Natural experiments (13%) used situational exposures and changes, such as loss of tree canopy from the EAB, to analyze changes in health outcomes. We classified observational studies into two groups: (a) longitudinal cohort or case-control (6%), and (b) cross-sectional studies (34%). Potential confounders addressed in such studies included data about prior conditions (such as asthma hospitalizations or chronic disease morbidity), demographic data derived from local or national census programs (such as income or poverty level, ethnicity, and population density) and physical environment attributes (such as renter occupied housing or land use type). The remaining studies utilized modelling (12%), or time series designs (7%). Modelling studies were limited to those that explicitly present environmental data or monitoring in association with health outcomes, often using regulatory or public health benchmarks, such as air particle concentrations or thermal comfort guidelines.

## 4. Results—Health Outcomes

Scoping reviews are as comprehensive, but much broader, than systematic reviews. Their utility is in the synthesis and categorization of the extent of existing research evidence in a given field in terms of focus, features, and volume [40]. Following the quality assessment, remaining studies were sorted across the three domains of the Markevych et al. framework [39]. Findings were further sorted into thematic subdomains, as introduced in Table 2.

### 4.1. Reducing Harm

#### 4.1.1. Air Pollutants and Respiratory Condition

Of the 14 total studies in this theme, 12 explored the role of urban trees and forests in reducing the harmful health effects of air pollution, including PM2.5, PM10, NO_2_, O_3_, SO_2_, cadmium, and benzopyrene. Studies in this subdomain were classified as either cross-sectional or modelling studies; no experimental, natural or quasi-experimental, longitudinal, cohort, and time series studies related to air pollutants or respiratory response were found. For the eight modelling studies, the health outcomes were all positive, such as reduced mortality (e.g., [41,42]), lower incidence of respiratory problems (e.g., [43,44,45]), and associated cost savings [46]. 

Two modelling studies assessed the amount of air pollutants removed/intercepted by trees and compared them to established air quality and health standards [47,48]. In the cross-sectional studies, several positive outcomes and a few neutral findings were reported. Positive outcomes included: lower prevalence of lung cancer associated with green space covered with trees within residential areas [49]; lower prevalence of asthma among children associated with greater street tree density [50]; and reduced asthma hospitalization associated with tree density during periods with high ambient pollutant concentrations [51]. Neutral findings included: no significant association between street tree density and asthma hospitalization among children [50]; and no significant association between tree density and asthma hospitalization during periods with lower ambient pollutant concentrations [51]. Of note, however, is a study by Lovasi et al. [52], which found that the prevalence of asthma among children did not appear to be related to tree canopy coverage, yet in some cases increased tree canopy coverage was positively associated with increased risks of asthma. 

Some studies indicated distinctive effects across vegetation types such as green space, gardens, trees, lawns, and bushes [49,51]. For example, Alcock et al. found that when ambient pollutant concentrations were lower, green space and gardens (but not trees) were associated with reductions in asthma hospitalization [51]. Whereas when ambient pollutant concentrations were higher, trees (but not green space and gardens) were associated with reductions in asthma hospitalization. Overall, urban trees and forests appear to remove a variety of air pollutants, which may in turn reduce some of the negative health outcomes associated with air pollution, although the magnitude of this benefit varies under different circumstances.

#### 4.1.2. Tree Pollen and Volatile Organic Compounds (VOCs)

We found 40 studies that evaluated tree pollen and VOCs emitted by trees, and their potential adverse effects on health such as the exacerbation of allergy, asthma, and rhinitis symptoms, and related behavior such as suicidal self-directed violence [53]. A range of study designs were employed with cross-sectional approaches being the most prevalent, followed by time series, longitudinal/cohort, modelling, and experimental studies (Table 2). Most studies found that higher pollen concentrations are associated with allergy exacerbation, which may lead to increased anti-allergy drug consumption [54,55,56,57] or hospital visits/admissions [58,59,60,61,62]. However, several studies noted that adverse health outcomes are not predicted solely by pollen concentrations as biophysical factors such as temperature, humidity, and ambient concentrations of air pollutants can produce synergies (e.g., [57,62,63,64,65,66]), and there is variable response associated with a person’s age [67].

Several time series studies found that pollen allergy prevalence is rising over time (e.g., [57,64]). Furthermore, while the pollen season typically occurs two to three months a year, climate change may lead to higher pollen concentrations and a longer pollen season (e.g., [68]). However, not all tree pollen has the same allergy-inducing potential; across various geographies some tree species were found to induce greater levels of pollen sensitization than others (e.g., [63,69,70,71,72,73,74,75,76]). For instance, olive and silver birch trees in Spain, and alder and Japanese cedar in China were found to have high allergenicity properties. Nonetheless, tree pollen has been found to cause fewer symptoms for some allergy sufferers than other types of aeroallergens such as indoor house dust mites, and other types of plant pollen such as grass and weed pollen (e.g., [71,77,78,79,80]). Allergy symptoms can also be exacerbated by co-sensitivity to tree pollen and other types of allergens (such as grass pollen; e.g., [81,82]). 

Two studies focused on the health impacts of VOCs [83,84], which are emitted naturally by trees. VOCs are an air quality concern because they are precursors to the formation of ozone [13,32]. Yet the effects of VOCs are not always negative, as one study found that smelling VOCs derived from *Cedrus deodara* can lead to increased relaxation and blood oxygenation with improvements to the respiratory or circulatory system, and decreased blood pressure [83]. Overall, tree pollen and VOCs have been associated with negative health outcomes, but these effects are not consistent across all tree species or urban living conditions, which suggests that these harmful effects can be reduced through tree selection and management practices.

#### 4.1.3. Ultraviolet Radiation (UVR)

Five studies investigated aspects of UVR exposure, a major risk factor for most skin cancers. Studies included the levels of sun exposure on children in schoolyards [85,86], solar radiation variability within different tree canopy structures [87], and personal solar erythemal UV exposure in tree shade [88,89]. Studies in this subdomain were classified as either natural/quasi-experimental or modelling studies (Table 2). Sun exposure was quantified using either dosimeters for measuring accumulated doses of UVR [90], or sensors for measuring irradiance. 

In general, the studies found that trees can reduce exposure to UVR, particularly bigger trees with a lower fraction of free sky (or sky view). However, studies also noted that visible shade is not the best indicator of UVR protection, as trees revealing a significant fraction of free sky may not provide adequate shade protection against UVB rays [87,89]. Other factors affecting the degree of protection provided by shade include individual behaviors (e.g., outdoor physical activity levels, clothing type) and the reflectivity of surrounding surfaces.

#### 4.1.4. Excess Heat and Thermal Comfort

There is strong evidence that trees reduce air and surface temperatures [91], but relatively few studies have explored the associated consequences for people’s health. We found a total of 17 studies investigating the association between urban trees and excess heat and thermal comfort. Within this subset, three studies focused on the relationship between trees and heat-related morbidity and mortality using longitudinal/cohort or modelling study designs. These studies found that: trees represent a risk-mitigating factor for heatstroke [92]; tree canopy cover was negatively correlated with heat-related ambulance calls during extreme heat events [93]; and increased vegetative cover was found to help offset projected increases in heat-related mortality for heat wave conditions in 2050 by 40 to 99% across three U.S. metropolitan regions [94]. Across this small group of studies, increased tree and vegetative cover were found to be beneficial in reducing the negative health effects of extreme heat.

The remaining 14 studies focused on thermal comfort using natural/quasi-experimental or modelling study designs (Table 2). Thermal comfort is typically quantified using various thermal indices such as changes in skin temperature (e.g., [95,96]), Physiologically Equivalent Temperature (PET) (e.g., [97,98,99,100]), the Universal Thermal Climate Index (UTCI) (e.g., [101,102]), and perceived thermal comfort (e.g., [95,98,103]). Among these 14 studies, PET, which expresses level of heat stress, was found to be the most commonly used index. Across these studies, actual and perceived thermal comfort were correlated. Many of these studies included field measurements of air temperature, humidity, wind speed, and other parameters, but rarely involved human subjects. Instead, they relied on established benchmarks of thermal conditions and human response. Overall, trees were found to help reduce the risks of heat-related morbidity and mortality and improve thermal comfort in outdoor spaces.

#### 4.1.5. Crime

We found six studies that focused on the relationship between trees and crime—a social, rather than environmental, source of harm. Studies within this subdomain were classified as either natural/quasi-experimental or cross-sectional (Table 2). These included studies of crime rates for both violent (e.g., murder, rape, robbery, and assault) and property crimes (e.g., burglary, theft, and arson) [104,105]. Generally, positive outcomes were associated with the presence of trees. Extensive tree mortality within neighborhoods, due to the EAB, was associated with increases in some types of crime in Cincinnati, U.S. [106]. In Philadelphia, U.S., the presence of tree cover was associated with reduced gun assaults [107], especially for young individuals in low-income urban areas. Troy et al. found that trees located on public property had a 40% greater crime reduction impact compared to trees on private property in Baltimore, U.S. [108]. There were some mixed findings; Donovan and Prestemon found that smaller, view-obstructing trees are associated with increased crime, while larger trees were associated with reduced crime in Portland, U.S. [109]. Overall, findings indicate that trees may reduce the incidence of various types of crime and possible influencing factors include the size, location, and health status of the trees.

### 4.2. Restoring Capacities

#### 4.2.1. Cognition and Attention Restoration

Research about the role of nature as a setting that supports cognition and attention restoration dates to the early 1990s [110]. Twelve studies considered the contribution of urban trees to mentally restorative experiences, either explicitly or as a substantial landscape element. All study designs were experiments or natural experiments. An additional study using fMRI scans (rather than the prevailing use of self-reported data) showed that forest settings prompted brain area activations related to involuntary attention [111], a theoretical precursor of cognitive restoration.

In the early 2000s, multiple studies optimized a natural experiment condition of randomized resident assignments within a Chicago public housing complex. Adult residents having nearby trees and lawns versus paved outdoor surfaces showed better attentional functioning and life management effectiveness [112] and reported less household aggression and violence [113]. Girls exhibited higher self-discipline scores (including concentration, impulse inhibition, and delay of gratification), but there were no effects for boys [114].

Randomized experiments of adults have explored the combined effect of physical activity in nature, finding that: cognitive function and mood state improved [115]; 55-minute walks enhanced cognitive performance [116]; and short sessions (15 minutes) of ‘forest bathing’ produced enhanced subjective feelings of vigor, recovery, and vitality [117]. However, not all findings were consistent. Perkins et al. compared the effects of 20-minute winter walks on a wooded trail, in a residential area, or in a parking lot, finding no difference in participants’ cognitive functioning; yet physical activity in all settings generated better short-term memory, as well as reduced fatigue, tension, anger, and depression [118].

Five studies examined specific features of restorative environments. Regarding evolutionary theory of how people engage with environments [119], Gatersleben et al. found that restorative natural environments had high levels of prospect and low levels of refuge traits [120]. For children, seasonal changes in tree foliage enhanced the perceived restorative quality of schoolyards [121]. Performance on attentional tests were improved when adults experienced streetscapes with trees, particularly when subjects were made more aware of the trees [122]. Similarly, Gathright et al. found that awareness of trees enhanced health benefits [123].

#### 4.2.2. Mental Health, Anxiety, and Mood

Out of the 15 studies that examined the effects of trees on mental health, anxiety, and mood, 12 studies used experiments or quasi-experiments, and three used cross-sectional designs. Several studies explored active and passive experiences of trees and nature on mental health, combining physiological and validated self-report measures. Within the shinrin-yoku (forest bathing) research (see Section 4.2.3), Morita et al. found that depression decreased and liveliness increased with forest immersion, with greater effects for subjects having higher initial stress levels [124]; exposure to forest settings produced lower measures of anxiety, depression, anger, confusion, and fatigue [125]; and for women, forest walking increased happiness more than walking in a gymnasium, with meditative walking in the forest being the most effective [126].

Two studies used neurophysiological measures to assess brain state. Near-infrared spectroscopy (NIRS) of adults revealed a more relaxed brain state, and lower self-reported “anger and hostility” and “total mood disturbance” for forest views [127]. MRI scans of adult city dwellers living close to a forest displayed an amygdala structure associated with better capacity to cope with stress [128]. These results align with findings from other studies that have used self-report data, which is the more common approach within this subdomain.

Five studies explored more specific contexts (e.g., workplaces and schools) and/or populations (e.g., children, adolescents, and tree climbers), primarily using self-report measures. A cross-sectional study found that having more forests and larger forest size in U.S. urban areas was associated with fewer days of mental health complaints [129]. Knowledge workers reported greater mental well-being improvements when having workplace views of trees and woodlands compared to time spent in open outdoor spaces [130]. Kim et al. found that larger and more treed areas within a neighborhood were positively correlated with Hispanic 4th and 5th graders’ reported health-related quality of life [131]. Studying adolescents from across a behavioral spectrum, Roe et al. found greater positive changes in energy, stress, hedonic tone, and anger at a forest school as compared to a conventional school, with a poor behavior group benefiting the most [132]. Participants in the TreeHab climbing therapy program showed decreased anger, fatigue and confusion, and increased vitality compared to recreational tree climbing, though results differed by tree species climbed and climber gender [133].

Many of the studies in this subdomain compared nature versus built environment stimulus to assess impact on mental health. However, three studies explored finer distinctions and considered exposure dosage. Electroencephalogram (EEG) response to simulations of forest tree stand density (30% to 100%) indicated more frontal brain activity when exposed to greater stand density, while lower stand density (from 30% to 50%) produced less brain activity, a more relaxed state, as well as reduced tension and fatigue [134]. Greater species richness may support mental well-being more than natural environments low in biodiversity, and even natural environments with low biodiversity may induce more positive affect than built environments [135]. Nonetheless, results are inconsistent. In a study comparing well-being effects of walks in wild and tended urban forests, changes in affect did not differ between treatment conditions, so physical activity may have been a stronger influence [136].

#### 4.2.3. Psychophysiological Stress

Twenty-five studies focused on the impacts of urban trees on psychophysiological stress. Twenty were experimental or natural/quasi-experimental, and five were observational designs. A central topic was shinrin-yoku (forest bathing), which emerged in the early 2000s, primarily in Japan and South Korea. Small sample experiments have compared health outcomes from experiences of forest versus urban settings, finding reduced prefrontal cerebral activity [137], lower salivary cortisol levels (an indicator of stress) [138], and suppressed sympathetic nervous activity (i.e., fight or flight response) accompanied by enhanced parasympathetic nervous activity (i.e., rest and digest state) [139]. A three-day field experiment reported positive results on a similar regime of measures [140] and was confirmed by a replicate study [141].

Following promising results for small-scale studies, forest bathing investigators expanded sampling and methods, utilizing combined physiological testing and self-reported mood states. Most studies compared subjects’ experiences in forest versus city areas, finding that nature influenced stress recovery and provided physiological benefits [142,143,144]. 

Some forest bathing studies delved deeper by focusing on specific subject traits such as age (e.g., [145,146]), personality types [147], and baseline health condition [148]. For age, findings include significant indications of stress reduction in young adults [145], middle-aged adults and the elderly [146], as well as elderly people with hypertension [149]. Toda et al. found that, for older men, walking in a woodland was a physical stressor compared to sitting in one’s office, but self-reported mental stress and mood improved following a forest walk [150]. Yet forest effects are not consistent; relaxed sitting with and without forest views generated similar positive cardiovascular outcomes in one study [151].

Four correlational studies within this subdomain have assessed larger study populations. Using state health survey data, Beyer et al. found that a 25% increase in neighborhood tree canopy was associated with a 1-point decrease on the 5-point Depression Anxiety and Stress Scales (DASS) instrument [152]. In another study, Egorov et al. found that reduced allostatic load, indicated by multiple biomarkers, was related to trees and other vegetation land cover in proximity to homes [153]. Survey studies have also found an inverse relationship between the total number of trees on a block and reported stress [154], and positive stress responses among visitors to broad leaved forests [155].

Nature dose-response is an important, practical concern [156], but results concerning urban trees are not yet conclusive. Using images with a specified range of street tree cover, one experimental study found that women displayed no stress recovery, but an inverted U-shaped dose response curve was observed for men, with slower rates of recovery for tree cover densities above 34% [157]. A similar study which used videos of variable street tree density (2% to 62%) found a linear association between density and self-reported stress recovery—irrespective of gender and age [158]. 

Studies have also examined varied tree and forest conditions in relation to stress recovery. Comparing visits to built-up versus green spaces, urban park and woodland settings had similar positive influences on cortisol measures, but perceived restorative effects were higher in a woodland [159]. Hauru et al. studied views of urban settings from inside a forest, finding higher perceived restorativeness when urban settings were less visible [160]. Applying prospect/refuge theory, more positive affects (e.g., attentiveness) and reduced negative affects (e.g., anger, aggression, and fear) were found in high prospect-low refuge environments [120]. This suggests that the spatial arrangements and configurations of trees, in addition to general nature content, can influence health response [161]. When comparing outcomes between urban streetscape and various urban forest settings—parkland, tended woodland, and wild woods—the urban forest settings promoted stronger stress recovery but no differences were found across the natural settings [162].

Workplaces are another study focus within this subdomain. Shin et al. found that self-reported job satisfaction and stress were improved with window views of forests [163]. A forest therapy program to address worker stress and burnout in healthcare and counseling service industries showed positive stress outcomes, particularly for clients who made more frequent visits [164]. However, questions remain about the distinct salutogenic effects of nature experience. A study of outdoor forest activity versus indoor handicraft interventions for workplace participants with high stress levels found that the well-being of all study participants improved, indicating that positive effects can also be found in non-natural environments that promote creative engagement [165].

#### 4.2.4. Clinical Outcomes

A total of 10 studies investigated the impact of exposure to trees or forests on the health of populations with clinical diagnoses. The majority used experimental study designs (Table 2) with a few case-control, longitudinal, and cross-sectional designs.

Studies of clinical populations with diagnosed mental health conditions found mainly positive results. For example, patients with major depression disorder [166,167] and exhaustion disorder [168] who participated in forest-based therapy showed improved outcomes including lower symptoms of depression, remission rates, mood and higher perceived restorativeness. In other studies, anti-depressant prescription rates were significantly lower with greater street tree density within London, England boroughs [169], and higher measures of tree canopy were associated with a lower prevalence of autism in California public school districts [170]. Mixed results were found following forest-based therapy for children with attention deficit hyperactivity disorder (ADHD) [171] and patients with exhaustion syndrome [172].

Three studies investigated physiological conditions in relation to exposure to urban trees and all found positive results. Significantly lower blood glucose, HbA1c, and blood pressure were noted in a longitudinal study of non-insulin dependent diabetic patients who participated in forest walks on nine occasions over a six-year time span [173]. An influential case-control study observed that patients with views of a tree through their window (versus view of a brick wall) had significantly shorter recovery times following gallbladder surgery [174]. Lastly, in all prefectures of Japan, the percentage of forest coverage was significantly inversely associated with the standardized mortality ratios for lung, breast, and uterine cancers in females, and prostate, kidney, and colon cancers in males after adjusting for smoking and socio-economic status [175]. This small group of studies found that exposure to trees among clinical populations produced mainly positive responses.

### 4.3. Building Capacities

#### 4.3.1. Birth Outcomes

Four studies investigated the association between trees and birth outcomes, of which three were longitudinal/cohort studies, and one cross-sectional study (Table 2). Various types of tree settings were investigated including street trees, tree cover, road-adjacent trees, or a combination thereof. Two studies reported positive findings including lower odds of preterm births in areas with more street trees [176], and a reduced number of small for gestational age (SGA) births with an increase in tree canopy cover (but not for preterm birth) [177]. One study found mixed results, with significant findings between tree cover and term birth weight in Austin, Texas, but not Portland, Oregon [178]. Positive effects have been found across varying buffers around the home, including 50 m [177], 100 m, 200 m, and 500 m [176]; and 1000 m [178]. Yet one study of major roads found no clear relationship between road-adjacent trees and the odds of low term birth weight and SGA births [179].

Potential pathways linking trees and birth outcomes include improvements to expectant mothers’ psychological health, increased rates of physical and social activity, and reduced exposure to air pollution (e.g., [176]). However, given the small number of studies, the relationship between trees and birth outcomes in urban areas remains unclear. The varied birth outcomes and types of tree settings examined also make it challenging to compare outcomes across studies. Questions remain about the importance of trees compared to other factors of influence such as maternal race/ethnicity [178]. Given the potential implications of adverse birth outcomes on health in later life stages, the relationship between trees and neonatal health warrants further exploration.

#### 4.3.2. Immune System

Six studies investigated the relationship between urban trees and immune function, using experimental study designs that involve visits to forests. Almost all of these studies were conducted by the same investigators across different areas of Japan. The number of subjects in each study ranged from 10 to 27. While almost all studies involved males only, one study involved all-females [180], and another involved children with gender unspecified [181]. 

Findings are consistently positive, indicating increased numbers of Natural Killer (NK) cells and NK activity, and reduced pro-inflammatory levels. It was found that increased NK activity can last more than seven days after a forest trip [180,182,183]. Among children with asthma or atopic dermatitis, Seo et al. found that a short visit to a forest resulted in significant improvements in various measures of disease severity and immunological effects [181]. It has been suggested that higher concentrations of phytoncides (aromatic VOCs released by trees), which are typically found in forest settings may contribute to increased NK activity [184]. Spending time in forest settings, even short visits, may promote healthier human immune systems, though underlying pathways are not completely understood [185].

#### 4.3.3. Active Living

Of the 19 studies that focused on physical activity, 18 found positive associations with exposure to urban trees. Within cross-sectional studies, objectively measured neighborhood tree cover was associated with higher levels of: commuting-related walking and cycling among adolescents [186]; self-reported recreational walking in adults [187]; levels of play among children [188]; and total free-time physical activity among students in grades 6 to 8 [189]. Perceptions of neighborhood tree cover were positively associated with: total physical activity for adults [190]; active transportation among adults with children living at home [191]; rates of recreational walking [192]; and predictive value for the longevity of urban senior residents across a five-year study [193]. One study, a natural experiment, found a negative association between EAB-induced tree loss and outdoor physical activity among adults in affected U.S. states [194].

Overall, urban trees appear to be related to increased physical activity across a range of populations, study designs, and geographic locations. Yet there were some mixed and negative findings, perhaps due to other factors such as culture, walkability, and concerns for safety. For instance, no significant association was found between physical activity and percent of neighborhood tree cover in adolescents in two German cities [195]. Urban U.S. census tracts with greater tree canopy density were associated with lower levels of commuting by walking or cycling [196]. Meanwhile, neighborhoods with the least tree coverage were associated with the highest amount of self-reported walking per week among middle-aged residents in Brisbane, Australia [197].

#### 4.3.4. Weight Status

All eight studies that considered urban trees and weight status were cross-sectional, and all found positive results. Weight status was most frequently characterized using Body Mass Index (BMI) thresholds for adults and percentiles for children [198]. Several studies found positive associations at the individual level between certain measures of tree exposure and reduced BMI, including: more tree patches and well-connected urban forests and trees [199]; greater proximity to forests [200]; and greater density of street trees [201,202]. At the population level, greater neighborhood tree canopy cover was associated with a lower prevalence of overweight and obese populations [203] and a 12% lower prevalence of obesity in preschool children [204]. BMI was found to be significantly lower in U.S. counties with higher per capita of forestland compared to rangeland, pastureland, or cropland [205]; and a higher percentage of normal BMI was found in U.S. Metropolitan Statistical Areas (MSAs) with greater forest edge density [206]. The reviewed articles provide evidence supporting a negative association between exposure to urban trees and unhealthy weight.

#### 4.3.5. Cardiovascular Function

Of the 16 articles exploring the impact of urban trees on cardiovascular health, most were experimental, with some cross-sectional or natural experiments (Table 2). Seven articles found that forest bathing improved cardiovascular function and related health outcomes among healthy participants, including: increased parasympathetic activity and reduced heart rate [207]; lower blood pressure [208,209]; and lower heart rate, diastolic blood pressure, and sympathetic activity [210]. Among participants with cardiovascular disease (CVD), exposure to forest settings was found to improve symptoms of hypertension more than urban settings, including: lower blood pressure and homocysteine (a CVD-related pathological factor) in elderly adults [211], and improved parasympathetic activity and reduced heart rate among middle-aged men [148,209]. However, another study of adults with hypertension found no significant differences in blood pressure between an experimental group receiving forest-based therapy and a control group [212].

Studies also investigated the association between urban trees and cardiovascular system-related outcomes. Mortality from CVD was found to be higher in EAB-infested U.S. states [213]. Sudden unexpected cardiac death was less likely in counties with a higher percentage of forest in North Carolina, U.S. [214]. Incidence of CVD was also found to be lower in neighborhoods with higher street tree density [215], and higher among women in EAB-infected U.S. counties [216]. However, no significant association was found between stress-related illnesses (i.e., heart attack and high blood pressure) and percent of tree canopy cover in MSAs in Texas, U.S. [217]. The majority of articles in this subdomain provide evidence for the positive influences of urban trees on cardiovascular health.

#### 4.3.6. Social Cohesion

Three articles investigated the relationship between urban trees and social cohesion, a measure of the degree to which residents feel a sense of connectedness, belonging, and trust [218]. In a study by Sullivan et al., more adults were observed performing social versus non-social activities in green versus barren neighborhood spaces in an inner-city public housing complex in Chicago, U.S. [219]. Another study in Baltimore, U.S., found that self-reported ratings of neighborhood social capital, connection, and association among individuals were positively associated with tree canopy cover, but not with two other measures of green space (i.e., access to recreational lands such as yards, and parks) [220]. Finally, Piff et al. found that participants staring up at large trees for one minute were significantly more likely to perform prosocial helping behaviors than a control group who stared up at large buildings [221]. While our collection of articles on trees and social cohesion is small, they show positive impacts of urban trees on the social well-being of individuals and communities.

## 5. Discussion

This scoping review reveals a large and rapidly growing body of research on urban trees and human health that is characterized by diverse study designs, pathways to health, and health outcomes. Organized under three overarching domains and subsequent research themes (or subdomains), our review provides readers with the opportunity to rapidly access research that is most relevant to their purposes (e.g., active living, weight status, or mental health). It also provides a transdisciplinary foundation for future research to help build more consistent and defensible knowledge that can inform approaches to improving community health, urban greening interventions, nature-based therapy, and clinical treatment. 

**Figure 2:**
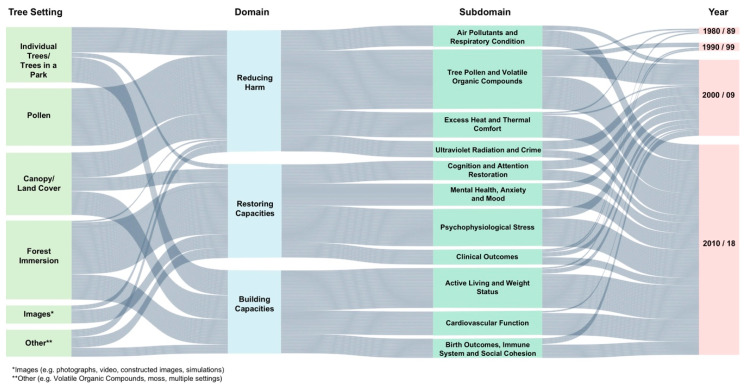
Scoping review of city trees and human health—synthesis of 201 studies.

As a high-level synthesis of the extent and diversity of this body of literature, Figure 2 presents an illustrative summary of the multiple relationships between urban trees and health, as well as the growth in range and volume of urban tree and health research over the past few decades (Supplementary Material Table S1: Citations and References for Table 2). The flows passing through each column illustrates the connections between tree settings (first column: Tree Setting), the biopsychosocial pathways identified by Markevych et al. (second column: Domain) [39], the subdomains that we interpreted based on the study results (third column: Research Theme), and the publication period by decade (fourth column: Year).

The studies examined exposure to multiple types of tree settings or influences (first column), including: individual trees, trees as pollen sources, trees in a park, canopy or land cover, forest immersion, and images or simulations of trees. ‘Other’ refers to VOCs, moss on trees, and tree loss due to EAB infestation. Across all three domains (second column), studies have incorporated the full range of tree settings. That being said, some types of tree settings appear more frequently in certain subdomains than in others. For example, forest immersion is associated predominantly with the Restoring Capacities domain, where a majority of the experimental studies involved visits to forested areas. Meanwhile, canopy/land cover—an approach to tree measurement that is often employed in epidemiological studies at larger geographical scales—was used more often in studies that fall under the Reducing Harm and Building Capacities domains.

Most of the 201 studies are classified within the Reducing Harm domain (82 studies), followed by Restoring Capacities (63), and Building Capacities (56), each containing multiple subdomains (third column). It should be noted that the volume of studies that represent any particular domain or subdomain does not equate to current strength of evidence. As shown in Table 2, study findings can vary in terms of positive, negative, or mixed and/or statistically insignificant outcomes. For themes with a greater number of studies (e.g., Active Living), there might be a greater mix of positive, negative, and mixed findings, which may be partly due to the diversity of research questions and methods used by researchers. We have observed studies focusing on different aspects of active living (e.g., walking, cycling, and levels of play among children), for example, or using different measures of physical activity (e.g., self-reported or measured data) and tree exposure (e.g., perceived or measured). This diversity has contributed to many unique insights, but also makes it challenging to aggregate overall findings into conclusive statements about strength of evidence.

Finally, Figure 2 also depicts the growth in research from the 1980s to the present time (fourth column). Three studies were published between 1980 and 1989, and five studies were published between 1990 and 1999. Almost all studies, or 96%, were published since 2000, with many published in 2010 or later (71% of 201 studies). Interest in some research topics has expanded faster than others over the same period. For example, research on stress reduction expanded from 7 studies published between 2000 and 2009 to 19 studies in 2010 to 2018, while clinical outcomes studies only increased from 3 to 5 during the same period.

### 5.1. Review Limitations

The purpose of this scoping review is to convey the full range of relationships between urban trees and human health based on existing quantitative research. It may not capture all current research as we limited the review to quantitative studies and English language publications. Future reviews that include qualitative studies and those in other languages could potentially expand understanding of health outcomes and generate further insights about causal relationships.

As in every review, it is possible that additional articles could have been retrieved by extension of search terms. Nevertheless, our search terms had a broad scope and it is unlikely the outcome of our search would have changed substantially by adding any additional terms.

We conducted a quality assessment on each article and included studies that have diverse yet robust methodological approaches in this scoping review. However, we did not assess the strength of evidence for each research theme as this is beyond the purpose of a typical scoping review, and more appropriate for systematic reviews with a smaller body of literature focused on more directly aligned research questions [33].

### 5.2. Research Needs

We found that the research on urban trees and health has evolved considerably from the 1980s to the present day, building on the momentum generated by promising findings from earlier studies (e.g., [112,139,174]), improvements in technology and data quality, and growing public interest and funding opportunities. This body of research covers multiple disciplines including public health, environmental health, urban planning, and urban forestry. Within this diversity, researchers have employed a diverse range of research questions, hypotheses, and methods that make it challenging to compare results. The population and geographic scales of studies were also diverse and not readily categorized.

More consistent research design and methods across studies, and replication, would be beneficial to enable cross-comparisons, including meta-analyses to generate more robust and conclusive evidence about phenomena and causality [222]. The development of shared research protocols or frameworks could also help build consistency across different types of research studies. For example, standardizing definitions in three key areas: (a) urban trees and forests, (b) health pathways and outcomes, and (c) site-specific characteristics, would facilitate better comparison across studies. 

While studies were conducted in cities around the world, they were largely focused in more industrialized, temperate climate regions, with a majority located in North America. The generalizability of research findings could be enhanced by repeating investigations across multiple climate zones, seasons, and settings, all of which can have variable influences on the impact of trees on people’s health—a recommendation that echoes Zeng and Dong who called for “complementary studies with longer periods, larger-scale surveys and during other seasons” [223] (p. 107). 

Additionally, the experimental and quasi-experimental studies collected for this review had small sample sizes. Out of the 57 experimental studies, over two-thirds of them had sample sizes of less than 50 participants, and most recruited only all-male subjects and young adults. Future urban tree and health research can be strengthened through larger sample sizes as well as better representation of residents in a particular location, with greater diversity across different ages, gender, and socio-economic conditions. Additional studies concerning other cultural or ethnographic settings or groups would confirm the salience of findings for more varied community or national populations. 

We observed that studies typically investigated short-term benefits such as improved blood pressure, stress reduction, and cognitive restoration. However, the durability and time-to-reverse of these benefits are less clear and would require longitudinal, time series, and other methods to assess sustained health outcomes. Future urban tree and health studies could also investigate how the duration and intermittence of people’s exposure patterns to trees can impact health outcomes. Other studies could explore what the consequences of short-term alleviation of symptoms following tree exposure might mean for patients with chronic diseases (such as improved blood pressure and cardiovascular function), and whether these short-term benefits could lead to reduced risk of developing chronic diseases later in life. 

Given the multiple potential mediating influences on people’s health, future research could investigate how aspects of peoples’ nature experiences, such as the health of trees, can influence other social determinants of health. For example, Jones found a negative, persistent relationship between EAB-infected trees and how people allocate leisure time in nature, which has consequences for physical activity [194]. Improving the understanding of the long-term effects of trees on people’s health would help strengthen the calls for investment in planting and nurturing trees.

Finally, future research could help improve understanding about the importance of site-specific characteristics on influencing health benefits. For example, Gilchrist et al. found that a different vegetation structure (e.g., the smooth ground layer, shrub layer, and canopy layer) can variably influence outcomes and suggested that the relationship of buildings to the landscape be considered at the outset of planning and design [130]. More detailed measures and reporting of study settings or exposure conditions, in addition to beneficiaries’ traits and temporal-spatial conditions, would help to better understand nature ‘dosage’ and response [156]. Standardized collection and reporting of functional form—such as the tree species, age, height, canopy density, physical location in relationship to nearby buildings, upkeep of the neighborhood, and density and size of tree stands—would also help inform urban forest planning and management to promote positive human health effects. 

While extensive discussions are underway about the implications of climate change on ecological systems and human health (e.g., [224,225,226,227]), we rarely encountered references to climate change in the 201 studies. Additional cross-disciplinary research is needed to better understand the risks of climate change on both human health and urban forests, including an exploration of how rapid environmental changes will affect tree health, and the consequent cascading impacts on human health, such as psychophysiological stress and heat-related illnesses and deaths. Such risks also highlight the importance of proactive urban forest management practices to secure and expand the ecological and health benefits of trees in the context of climate change mitigation and adaptation [228].

## 6. Conclusions

Our findings support the growing public recognition of urban trees as an essential component of health-supportive environments (e.g., [229,230]). It is important, however, not to overstate the current evidence. For instance, the effects of trees vary by person and may not always be beneficial, such as the potential for tree pollen to exacerbate allergy conditions. Additionally, the benefits of trees are affected by the health status of trees and forests. For instance, Donovan et al. found that EAB-infested trees have been associated with adverse health outcomes [213,216]. Salmond et al. found that VOCs are emitted when trees are under stress (e.g., due to drought, heat, and pests) [32]. There is variable response to tree shading within streetscape conditions [231] and across winter and summer seasons [232]. These site-specific considerations suggest that integrated and proactive design and management of urban trees could double as an experimental approach to human health research and minimize potential adverse impacts. 

One observation of note is the prevalence of articles emerging from the PubMed search. Reactions to pollen, such as allergenicity and asthma, were prominent. Fewer articles highlighting salutogenic benefits of urban trees appeared from the PubMed search, perhaps leading to less awareness among public health and medical professionals about the diversity of tree-based health determinants. 

Implementing trees as a health intervention in a community is a long-term, even multi-decade, investment. Urban forestry and health professionals could work together to better integrate human health outcomes into urban forestry best practices more directly by actively translating the full scope of science into practice [233]. This could involve increased collaboration between health and environmental professionals in developing evidence-based resources such as tree planting guidelines that support positive human health outcomes, while considering site-specific characteristics and a range of population needs (e.g., to support active living across all ages). Greater collaboration between health and environmental professionals in the design process could also achieve the goals of co-designing for co-benefits. For example, trees that are planted with the primary goal of improving stormwater management could also be configured to optimize a range of additional positive health outcomes such as stress reduction and social cohesion [234]. 

Overall, we have found that exposure to trees is associated with multiple health benefits. Underlying this relationship is the importance of access. Studies have found that there are often disparities in distribution of trees in urban areas with greater tree density being found in neighborhoods having higher household incomes (e.g., [235,236]), which may in turn exacerbate existing socio-demographic health inequities. For example, people who may not have sufficient resources to operate air conditioning in their homes may also live in neighborhoods that lack the cooling benefits of urban trees, thereby compounding their vulnerability to extreme heat events [236]. Adopting a health equity lens in the planning and management of urban forests can ensure a more equitable distribution of trees across towns and cities and provide residents with access to the health benefits of trees. 

Identifying who is vulnerable to different health outcomes and where they live and work can also inform the development of more targeted strategies or interventions (e.g., urban greening and nature-based therapy) to maximize health benefits. When community members are involved in the development of these urban greening programs, additional benefits can be gained, such as increased civic engagement and social interaction [237]. Most towns and cities face many competing funding priorities. Our review suggests that urban trees may be a low-cost policy intervention that addresses multiple environmental and human health co-benefits. Investing in the proactive planning and management of urban trees can pay human well-being and economic dividends.

## Figures and Tables

**Table 1:** Search terms used to access publications concerning urban trees and human health.

Database	Search Terms
Used in All Searches	AND/OR(tree, trees, treed, sapling*, seedling*, wooded, canop*), OR(urban*, suburban*, cities, city, town*, neighbourhood*, neighborhood*, metropol*, borough*), OR(human*, person*, people*, man, men?, wom?n, child*, infan*, toddler*, newborn*, neonat*, baby, babies, adolecen*, teenage*, preteen*, preadolescen*, premenarch*, pre menarch*, adult*, elderly, seniors), OR(health, epidemiology, quality of life), limit to English language)
	**Additional Search Terms**
**Embase**	OR(urban area, urban population, city planning), OR(general aspects of diseases, mental disease, physical disease, mortality risk, epidemiological monitoring, epidemiological data, genotoxicity, genetic damage, mutagenic activity, mutagenicity, postnatal development, toxicity, biological functions, environmental health, environmental stress, hospital admission)
**Ovid Medline**	OR(urban population, city planning, environmental design, urban renewal), OR(mental disorders, epidemiological monitoring, growth and development, hospitalization)
**PsycINFO**	OR(urban planning, urban environments), OR(community health, health attitudes, health behavior, health complaints, health disparities, public health services, well being, international classification of diseases, mental disorders, psychodiagnosis, environmental psychology, ecological psychology, death and dying, life satisfaction, facility admission, hospitalization, psychiatric hospital admission, health care seeking behavior)
**PubMed**	OR(urban population, city planning, environment design, urban renewal), OR(mental disorders, epidemiological monitoring, growth and development, hospitalization)

Legend: * indicates truncated term to access word derivatives; ? indicates wildcard to access spelling variations for term.

**Table 2:** Summary of studies in urban trees and human health scoping review, sorted by health outcomes domains and study design. (narrative provides details of thematic analysis; citations and references are in Supplementary Material, Table S1).

Domain/Subdomain	Study Count	Experiment	Natural/Quasi-Experiment	Longitudinal/Cohort	Cross Sectional	Modelling	Time Series
**4.1. Reducing Harm**	**82**						
4.1.1 Air Pollutants and Respiratory Condition	14				●●◐◐◐ ○	●●●●● ●●●	
4.1.2 Tree Pollen and VOCs	40	◐	◐	○○○	◐◐◐◐◐ ◐◐◐◐◐ ◐◐◐◐ ○○○○	○○○	◐◐◐◐ ○○○○○ ○○○○○
4.1.3 Ultraviolet Radiation	5		●●			●◐◐	
4.1.4 Excess Heat and Thermal Comfort	17		●●●●●	●●		●●●●● ●●●●◐	
4.1.5 Crime	6		●● ◐		●●◐		
**4.2. Restoring Capacities**	**63**						
4.2.1 Cognition and Attention Restoration	13	●●●●● ●◐◐	●●●◐		●		
4.2.2 Mental Health, Anxiety and Mood	15	●●●●● ●●●● ◐◐	●		●◐◐		
4.2.3 Psychophysiological Stress	25	●●●●● ●●●●● ●●●● ◐◐◐◐	●●	◐	●●●●		
4.2.4 Clinical Outcomes	10	●●● ◐	◐	●●	●●●		
**4.3. Building Capacities**	**56**						
4.3.1 Birth Outcomes	4			◐◐◐	●		
4.3.2 Immune System	6	●●●●● ●					
4.3.3 Active Living	19		●●		●●●●● ●●●●● ◐◐◐◐◐ ○○		
4.3.4 Weight Status	8				●●●●● ●●●		
4.3.5 Cardiovascular Function	16	●●●●● ●●◐◐	●●●◐		●◐◐		
4.3.6 Social Cohesion	3		●		●●		
**Total Studies**	**201**	**57**	**26**	**12**	**69**	**24**	**13**

Legend: Each circle represents one study - ● = positive outcome, ◐ = mixed and/or insignificant outcome, ○ = negative outcome.

## Supplementary Materials

The following is available online at https://www.mdpi.com/1660-4601/17/12/4371/s1, Table S1: Citations and References for Table 2.

## References

[1] Pearlmutter D., Calfapietra C., Samson R., O’Brien L., Ostoić S.K., Sanesi G., del Amo R.A. (2017). The Urban Forest: Cultivating Green Infrastructure for People and the Environment.

[2] Tiwary A., Williams I.D., Heidrich O., Namdeo A., Bandaru V., Calfapietra C. (2016). Development of multi-functional streetscape green infrastructure using a performance index approach. Environ. Pollut..

[3] Li F., Liu X., Zhang X., Zhao D., Liu H., Zhou C., Wang R. (2017). Urban ecological infrastructure: An integrated network for ecosystem services and sustainable urban systems. J. Clean. Prod..

[4] O’Brien L., De Vreese R., Kern M., Sievänen T., Stojanova B., Atmiş E. (2017). Cultural ecosystem benefits of urban and peri-urban green infrastructure across different European countries. Urban For. Urban Green..

[5] Tyrväinen L., Pauleit S., Seeland K., de Vries S., Konijnendijk C., Nilsson K., Randrup T., Schipperijn J. (2005). Benefits and uses of urban forests and trees. Urban Forests and Trees.

[6] Konijnendijk C.C., Ricard R.M., Kenney A., Randrup T.B. (2006). Defining urban forestry—A comparative perspective of North America and Europe. Urban For. Urban Green..

[7] Dwyer J.F., McPherson E.G., Schroeder H.W., Rowntree R.A. (1992). Assessing the benefits and costs of the urban forest. J. Arboric..

[8] Chen W.Y., Jim C.Y., Carreiro M.M., Song Y.-C., Wu J. (2008). Assessment and valuation of the ecosystem services provided by urban forests. Ecology, Planning, and Management of Urban Forests: International Perspectives.

[9] Dobbs C., Escobedo F.J., Zipperer W.C. (2011). A framework for developing urban forest ecosystem services and goods indicators. Landsc. Urban Plan..

[10] Roy S., Byrne J., Pickering C. (2012). A systematic quantitative review of urban tree benefits, costs, and assessment methods across cities in different climatic zones. Urban For. Urban Green..

[11] Davies H., Doick K., Handley P., O’Brien L., Wilson J. (2017). Delivery of Ecosystem Services By Urban Forests.

[12] Nowak D.J., Crane D.E. (2002). Carbon storage and sequestration by urban trees in the USA. Environ. Pollut..

[13] Nowak D.J., Crane D.E., Stevens J.C. (2006). Air pollution removal by urban trees and shrubs in the United States. Urban For. Urban Green..

[14] Nowak D.J., Hoehn R., Crane D.E. (2007). Oxygen production by urban trees in the United States. Arboric. Urban For..

[15] Xiao Q., McPherson E.G. (2002). Rainfall interception by Santa Monica’s municipal urban forest. Urban Ecosyst..

[16] Oke T.R. (1989). The micrometeorology of the urban forest. Philos. Trans. R. Soc. Lond. B Biol. Sci..

[17] Bowler D.E., Buyung-Ali L., Knight T.M., Pullin A.S. (2010). Urban greening to cool towns and cities: A systematic review of the empirical evidence. Landsc. Urban Plan..

[18] Bowler D.E., Buyung-Ali L.M., Knight T.M., Pullin A.S. (2010). A systematic review of evidence for the added benefits to health of exposure to natural environments. BMC Public Health.

[19] Hartig T., Mitchell R., de Vries S., Frumkin H. (2014). Nature and health. Annu. Rev. Public Health.

[20] Berto R. (2014). The role of nature in coping with psycho-physiological stress: A literature review on restorativeness. Behav. Sci..

[21] Kuo M. (2015). How might contact with nature promote human health? Promising mechanisms and a possible central pathway. Front. Psychol..

[22] James P., Banay R.F., Hart J.E., Laden F. (2015). A review of the health benefits of greenness. Curr. Epidemiol. Rep..

[23] Lee A.C.K., Maheswaran R. (2011). The health benefits of urban green spaces: A review of the evidence. J. Public Health.

[24] Lachowycz K., Jones A.P. (2011). Greenspace and obesity: A systematic review of the evidence. Obes. Rev..

[25] Kabisch N., Qureshi S., Haase D. (2015). Human–environment interactions in urban green spaces—A systematic review of contemporary issues and prospects for future research. Environ. Impact Assess. Rev..

[26] Gascon M., Triguero-Mas M., Martínez D., Dadvand P., Rojas-Rueda D., Plasència A., Nieuwenhuijsen M.J. (2016). Residential green spaces and mortality: A systematic review. Environ. Int..

[27] Bogar S., Beyer K.M. (2016). Green space, violence, and crime: A systematic review. Trauma Violence Abuse.

[28] Hunter R.F., Cleland C., Cleary A., Droomers M., Wheeler B.W., Sinnett D., Nieuwenhuijsen M.J., Braubach M. (2019). Environmental, health, wellbeing, social and equity effects of urban green space interventions: A meta-narrative evidence synthesis. Environ. Int..

[29] Fong K.C., Hart J.E., James P. (2018). A review of epidemiologic studies on greenness and health: Updated literature through 2017. Curr. Environ. Health Rep..

[30] House E., O’Connor C., Wolf K.L., Israel J., Reynolds T. (2016). Outside Our Doors: The Benefits of Cities Where People and Nature Thrive.

[31] Willamette Partnership, Oregon Public Health Institute (2018). Green Infrastructure & Health Guide.

[32] Salmond J.A., Tadaki M., Vardoulakis S., Arbuthnott K., Coutts A., Demuzere M., Dirks K.N., Heaviside C., Lim S., Macintyre H. (2016). Health and climate related ecosystem services provided by street trees in the urban environment. Environ. Health.

[33] Pham M.T., Rajić A., Greig J.D., Sargeant J.M., Papadopoulos A., McEwen S.A. (2014). A scoping review of scoping reviews: Advancing the approach and enhancing the consistency. Res. Synth. Methods.

[34] Peters M.D.J., Godfrey C.M., Khalil H., McInerney P., Parker D., Soares C.B. (2015). Guidance for conducting systematic scoping reviews. Int. J. Evid. Based Healthc..

[35] Khalil H., Peters M., Godfrey C.M., McInerney P., Soares C.B., Parker D. (2016). An evidence-based approach to scoping reviews. Worldviews Evid. Based Nurs..

[36] Nowak D.J., Greenfield E.J. (2018). Declining urban and community tree cover in the United States. Urban For. Urban Green..

[37] Tricco A.C., Lillie E., Zarin W., O’Brien K.K., Colquhoun H., Levac D., Moher D., Peters M.D.J., Horsley T., Weeks L. (2018). PRISMA extension for scoping reviews (PRISMA-ScR): Checklist and explanation. Ann. Intern. Med..

[38] McMaster Evidence Review and Synthesis Team Effective Public Health Practice Project (EPHPP): Quality Assessment Tool for Quantitative Studies. https://merst.ca/ephpp/.

[39] Markevych I., Schoierer J., Hartig T., Chudnovsky A., Hystad P., Dzhambov A.M., de Vries S., Triguero-Mas M., Brauer M., Nieuwenhuijsen M.J. (2017). Exploring pathways linking greenspace to health: Theoretical and methodological guidance. Environ. Res..

[40] Grant M.J., Booth A. (2009). A typology of reviews: An analysis of 14 review types and associated methodologies. Health Inf. Libr. J..

[41] Tiwary A., Sinnett D., Peachey C., Chalabi Z., Vardoulakis S., Fletcher T., Leonardi G., Grundy C., Azapagic A., Hutchings T.R. (2009). An integrated tool to assess the role of new planting in PM10 capture and the human health benefits: A case study in London. Environ. Pollut..

[42] Nowak D.J., Hirabayashi S., Bodine A., Hoehn R. (2013). Modeled PM2.5 removal by trees in ten U.S. cities and associated health effects. Environ. Pollut..

[43] Rao M., George L.A., Rosenstiel T.N., Shandas V., Dinno A. (2014). Assessing the relationship among urban trees, nitrogen dioxide, and respiratory health. Environ. Pollut..

[44] Rao M., George L.A., Shandas V., Rosenstiel T.N. (2017). Assessing the potential of land use modification to mitigate ambient NO2 and its consequences for respiratory health. Int. J. Environ. Res. Public Health.

[45] Hirabayashi S., Nowak D.J. (2016). Comprehensive national database of tree effects on air quality and human health in the United States. Environ. Pollut..

[46] Nowak D.J., Hirabayashi S., Bodine A., Greenfield E. (2014). Tree and forest effects on air quality and human health in the United States. Environ. Pollut..

[47] Donovan G.H., Jovan S.E., Gatziolis D., Burstyn I., Michael Y.L., Amacher M.C., Monleon V.J. (2016). Using an epiphytic moss to identify previously unknown sources of atmospheric cadmium pollution. Sci. Total Environ..

[48] Ratola N., Jiménez-Guerrero P. (2017). Modelling benzo[a]pyrene in air and vegetation for different land uses and assessment of increased health risk in the Iberian Peninsula. Environ. Sci. Pollut. Res..

[49] Wang L., Zhao X., Xu W., Tang J., Jiang X. (2016). Correlation analysis of lung cancer and urban spatial factor: Based on survey in Shanghai. J. Thorac. Dis..

[50] Lovasi G.S., Quinn J.W., Neckerman K.M., Perzanowski M.S., Rundle A. (2008). Children living in areas with more street trees have lower prevalence of asthma. J. Epidemiol. Community Health.

[51] Alcock I., White M., Cherrie M., Wheeler B., Taylor J., McInnes R., Otte im Kampe E., Vardoulakis S., Sarran C., Soyiri I. (2017). Land cover and air pollution are associated with asthma hospitalisations: A cross-sectional study. Environ. Int..

[52] Lovasi G.S., O’Neil-Dunne J.P.M., Lu J.W.T., Sheehan D., Perzanowski M.S., MacFaden S.W., King K.L., Matte T., Miller R.L., Hoepner L.A. (2013). Urban tree canopy and asthma, wheeze, rhinitis, and allergic sensitization to tree pollen in a New York City birth cohort. Environ. Health Perspect..

[53] Jeon-Slaughter H., Claassen C.A., Khan D.A., Mihalakos P., Lee K.B., Brown E.S. (2016). Temporal association between nonfatal self-directed violence and tree and grass pollen counts. J. Clin. Psychiatry.

[54] Alcázar P., Cariñanos P., De Castro C., Guerra F., Moreno C., Dominguez-Vilches E., Galán C. (2004). Airborne plane-tree (*Platanus hispanica*) pollen distribution in the city of Córdoba, southwestern Spain, and possible implications on pollen allergy. J. Investig. Allergol. Clin. Immunol..

[55] Sheffield P.E., Weinberger K.R., Ito K., Matte T.D., Mathes R.W., Robinson G.S., Kinney P.L. (2011). The association of tree pollen concentration peaks and allergy medication sales in New York City: 2003–2008. ISRN Allergy.

[56] Motreff Y., Golliot F., Calleja M., Le Pape A., Fuhrman C., Farrera I., Plaisant I. (2014). Short-term effect of pollen exposure on drug consumption for allergic rhinitis and conjunctivitis. Aerobiologia.

[57] Caillaud D.M., Martin S., Ségala C., Vidal P., Lecadet J., Pellier S., Rouzaire P., Tridon A., Evrard B. (2015). Airborne pollen levels and drug consumption for seasonal allergic rhinoconjunctivitis: A 10-year study in France. Allergy.

[58] Cakmak S., Dales R.E., Coates F. (2012). Does air pollution increase the effect of aeroallergens on hospitalization for asthma?. J. Allergy Clin. Immunol..

[59] Jariwala S.P., Kurada S., Moday H., Thanjan A., Bastone L., Khananashvili M., Fodeman J., Hudes G., Rosenstreich D. (2011). Association between tree pollen counts and asthma ED visits in a high-density urban center. J. Asthma.

[60] Jariwala S., Toh J., Shum M., de Vos G., Zou K., Sindher S., Patel P., Geevarghese A., Tavdy A., Rosenstreich D. (2014). The association between asthma-related emergency department visits and pollen and mold spore concentrations in the Bronx, 2001–2008. J. Asthma.

[61] Weichenthal S., Lavigne E., Villeneuve P.J., Reeves F. (2016). airborne pollen concentrations and emergency room visits for myocardial infarction: A multicity case-crossover study in Ontario, Canada. Am. J. Epidemiol..

[62] Osborne N.J., Alcock I., Wheeler B.W., Hajat S., Sarran C., Clewlow Y., McInnes R.N., Hemming D., White M., Vardoulakis S. (2017). Pollen exposure and hospitalization due to asthma exacerbations: Daily time series in a European city. Int. J. Biometeorol..

[63] Dales R.E., Cakmak S., Judek S., Dann T., Coates F., Brook J.R., Burnett R.T. (2004). Influence of outdoor aeroallergens on hospitalization for asthma in Canada. J. Allergy Clin. Immunol..

[64] Sánchez-Mesa J.A., Serrano P., Cariñanos P., Prieto-Baena J.C., Moreno C., Guerra F., Galan C. (2005). Pollen allergy in Cordoba city: Frequency of sensitization and relation with antihistamine sales. J. Investig. Allergol. Clin. Immunol..

[65] Diaz de la Guardia C., Alba F., de Linares C., Nieto-Lugilde D., López Caballero J. (2006). Aerobiological and allergenic analysis of Cupressaceae pollen in Granada (southern Spain). J. Investig. Allergol. Clin. Immunol..

[66] May L., Carim M., Yadav K. (2011). Adult asthma exacerbations and environmental triggers: A retrospective review of ED visits using an electronic medical record. Am. J. Emerg. Med..

[67] Sheehan W.J., Rangsithienchai P.A., Baxi S.N., Gardynski A., Bharmanee A., Israel E., Phipatanakul W. (2010). Age-specific prevalence of outdoor and indoor aeroallergen sensitization in Boston. Clin. Pediatr..

[68] Lake I.R., Jones N.R., Agnew M., Goodess C.M., Giorgi F., Hamaoui-Laguel L., Semenov M.A., Solomon F., Storkey J., Vautard R. (2017). Climate change and future pollen allergy in Europe. Environ. Health Perspect..

[69] Lin R.Y., Clauss A.E., Bennett E.S. (2002). Hypersensitivity to common tree pollens in New York City patients. Allergy Asthma Proc..

[70] Dales R.E., Cakmak S., Judek S., Coates F. (2008). Tree pollen and hospitalization for asthma in urban Canada. Int. Arch. Allergy Immunol..

[71] Loureiro G., Rabaça M.A., Blanco B., Andrade S., Chieira C., Pereira C. (2005). Urban versus rural environment--any differences in aeroallergens sensitization in an allergic population of Cova da Beira, Portugal?. Eur. Ann. Allergy Clin. Immunol..

[72] Díaz J., Linares C., Tobías A. (2007). Short-term effects of pollen species on hospital admissions in the City of Madrid in terms of specific causes and age. Aerobiologia.

[73] Fuhrman C., Sarter H., Thibaudon M., Delmas M.-C., Zeghnoun A., Lecadet J., Caillaud D. (2007). Short-term effect of pollen exposure on antiallergic drug consumption. Ann. Allergy. Asthma. Immunol..

[74] Darrow L.A., Hess J., Rogers C.A., Tolbert P.E., Klein M., Sarnat S.E. (2012). Ambient pollen concentrations and emergency department visits for asthma and wheeze. J. Allergy Clin. Immunol..

[75] Puiggròs A., Muñoz-Cano R., Reig A.R., Raga E., Belmonte J., Valero A. (2015). Prevalence of sensitization to pollen from trees planted in Barcelona City. J. Investig. Allergol. Clin. Immunol..

[76] Cariñanos P., Adinolfi C., Díaz de la Guardia C., De Linares C., Casares-Porcel M. (2016). characterization of allergen emission sources in urban areas. J. Environ. Qual..

[77] Sam C.K., Soon S.C., Liam C.K., Padmaja K., Cheng H.M. (1998). An investigation of aeroallergens affecting urban Malaysian asthmatics. Asian Pac. J. Allergy Immunol..

[78] Harmanci E., Metintas E. (2000). The type of sensitization to pollens in allergic patients in Eskisehir (Anatolia), Turkey. Allergol. Immunopathol..

[79] Akerman M., Valentine-Maher S., Rao M., Taningco G., Khan R., Tuysugoglu G., Joks R. (2003). Allergen sensitivity and asthma severity at an inner city asthma center. J. Asthma.

[80] Kim D.H., Park Y.-S., Ji Jang H., Kim J.H., Lim D.H. (2016). Prevalence and allergen of allergic rhinitis in Korean children. Am. J. Rhinol. Allergy.

[81] Vieira F., Ferreira E., Cruz A. (1998). Grass allergy increases the risk of tree pollen sensitization: A warning to urban planners. J. Allergy Clin. Immunol..

[82] Celik G., Mungan D., Pinar M., Misirligil Z. (2005). Poplar pollen-related allergy in Ankara, Turkey: How important for patients living in a city with high pollen load?. Allergy Asthma Proc..

[83] Song X., Li H., Li C., Xu J., Hu D. (2016). Effects of VOCs from leaves of *Acer truncatum* Bunge and *Cedrus deodara* on human physiology and psychology. Urban For. Urban Green..

[84] Ren Y., Qu Z., Du Y., Xu R., Ma D., Yang G., Shi Y., Fan X., Tani A., Guo P. (2017). Air quality and health effects of biogenic volatile organic compounds emissions from urban green spaces and the mitigation strategies. Environ. Pollut..

[85] Boldeman C., Dal H., Wester U. (2004). Swedish pre-school children’s UVR exposure—A comparison between two outdoor environments. Photodermatol. Photoimmunol. Photomed..

[86] Boldemann C., Blennow M., Dal H., Mårtensson F., Raustorp A., Yuen K., Wester U. (2006). Impact of preschool environment upon children’s physical activity and sun exposure. Prev. Med..

[87] Heisler G.M., Grant R.H., Gao W. (2003). Individual- and scattered-tree influences on ultraviolet irradiance. Agric. For. Meteorol..

[88] Parisi A.V., Kimlin M.G., Wong J.C.F., Lester R., Turnbull D. (2000). Reduction in the personal annual solar erythemal ultraviolet exposure provided by Australian gum trees. Radiat. Prot. Dosimetry.

[89] Parisi A.V., Kimlin M.G., Wong J.C.F., Wilson M. (2000). Personal exposure distribution of solar erythemal ultraviolet radiation in tree shade over summer. Phys. Med. Biol..

[90] Thieden E., Philipsen P.A., Heydenreich J., Wulf H.C. (2004). UV radiation exposure related to age, sex, occupation, and sun behavior based on time-stamped personal dosimeter readings. Arch. Dermatol..

[91] Livesley S.J., McPherson E.G., Calfapietra C. (2016). The urban forest and ecosystem services: Impacts on urban water, heat, and pollution cycles at the tree, street, and city scale. J. Environ. Qual..

[92] Kilbourne E.M. (1982). Risk factors for heatstroke: A case-control study. JAMA.

[93] Graham D.A., Vanos J.K., Kenny N.A., Brown R.D. (2016). The relationship between neighbourhood tree canopy cover and heat-related ambulance calls during extreme heat events in Toronto, Canada. Urban For. Urban Green..

[94] Stone B., Vargo J., Liu P., Habeeb D., DeLucia A., Trail M., Hu Y., Russell A. (2014). Avoided heat-related mortality through climate adaptation strategies in three US cities. PLoS ONE.

[95] Jeong M.-A., Park S., Song G.-S. (2016). Comparison of human thermal responses between the urban forest area and the central building district in Seoul, Korea. Urban For. Urban Green..

[96] Song G.-S., Jeong M.-A. (2016). Morphology of pedestrian roads and thermal responses during summer, in the urban area of Bucheon City, Korea. Int. J. Biometeorol..

[97] Johansson E., Emmanuel R. (2006). The influence of urban design on outdoor thermal comfort in the hot, humid city of Colombo, Sri Lanka. Int. J. Biometeorol..

[98] Klemm W., Heusinkveld B.G., Lenzholzer S., Jacobs M.H., Van Hove B. (2015). Psychological and physical impact of urban green spaces on outdoor thermal comfort during summertime in The Netherlands. Build. Environ..

[99] Lee H., Mayer H., Chen L. (2016). Contribution of trees and grasslands to the mitigation of human heat stress in a residential district of Freiburg, southwest Germany. Landsc. Urban Plan..

[100] Kántor N., Chen L., Gál C.V. (2018). Human-biometeorological significance of shading in urban public spaces—Summertime measurements in Pécs, Hungary. Landsc. Urban Plan..

[101] Wu Z., Kong F., Wang Y., Sun R., Chen L. (2016). The impact of greenspace on thermal comfort in a residential quarter of Beijing, China. Int. J. Environ. Res. Public Health.

[102] Milošević D.D., Bajšanski I.V., Savić S.M. (2017). Influence of changing trees locations on thermal comfort on street parking lot and footways. Urban For. Urban Green..

[103] Lin T.-P., Tsai K.-T., Liao C.-C., Huang Y.-C. (2013). Effects of thermal comfort and adaptation on park attendance regarding different shading levels and activity types. Build. Environ..

[104] Kuo F.E., Sullivan W.C. (2001). Environment and crime in the inner city: Does vegetation reduce crime?. Environ. Behav..

[105] Gilstad-Hayden K., Wallace L.R., Carroll-Scott A., Meyer S.R., Barbo S., Murphy-Dunning C., Ickovics J.R. (2015). Greater tree canopy cover is associated with lower rates of both violent and property crime in New Haven, CT. Landsc. Urban Plan..

[106] Kondo M.C., Han S., Donovan G.H., MacDonald J.M. (2017). The association between urban trees and crime: Evidence from the spread of the emerald ash borer in Cincinnati. Landsc. Urban Plan..

[107] Kondo M.C., South E.C., Branas C.C., Richmond T.S., Wiebe D.J. (2017). The association between urban tree cover and gun assault: A case-control and case-crossover study. Am. J. Epidemiol..

[108] Troy A., Morgan Grove J., O’Neil-Dunne J. (2012). The relationship between tree canopy and crime rates across an urban–rural gradient in the greater Baltimore region. Landsc. Urban Plan..

[109] Donovan G.H., Prestemon J.P. (2012). The effect of trees on crime in Portland, Oregon. Environ. Behav..

[110] Kaplan S., Peterson C. (1993). Health and environment: A psychological analysis. Landsc. Urban Plan..

[111] Martínez-Soto J., Gonzales-Santos L., Pasaye E., Barrios F.A. (2013). Exploration of neural correlates of restorative environment exposure through functional magnetic resonance. Intell. Build. Int..

[112] Kuo F.E. (2001). Coping with poverty: Impacts of environment and attention in the inner city. Environ. Behav..

[113] Kuo F.E., Sullivan W.C. (2001). Aggression and violence in the inner city: Effects of environment via mental fatigue. Environ. Behav..

[114] Taylor A.F., Kuo F.E., Sullivan W.C. (2002). Views of nature and self-discipline: Evidence from inner city children. J. Environ. Psychol..

[115] Shin W.S., Shin C.S., Yeoun P.S., Kim J.J. (2011). The influence of interaction with forest on cognitive function. Scand. J. For. Res..

[116] Berman M.G., Jonides J., Kaplan S. (2008). The cognitive benefits of interacting with nature. Psychol. Sci..

[117] Takayama N., Korpela K., Lee J., Morikawa T., Tsunetsugu Y., Park B.-J., Li Q., Tyrväinen L., Miyazaki Y., Kagawa T. (2014). Emotional, restorative and vitalizing effects of forest and urban environments at four sites in Japan. Int. J. Environ. Res. Public Health.

[118] Perkins S., Searight H.R., Ratwik S. (2011). Walking in a natural winter setting to relieve attention fatigue: A pilot study. Psychology.

[119] Appleton J. (1975). The Experience of Landscape.

[120] Gatersleben B., Andrews M. (2013). When walking in nature is not restorative—The role of prospect and refuge. Health Place.

[121] Paddle E., Gilliland J. (2016). Orange is the new green: Exploring the restorative capacity of seasonal foliage in schoolyard trees. Int. J. Environ. Res. Public Health.

[122] Lin Y.-H., Tsai C.-C., Sullivan W.C., Chang P.-J., Chang C.-Y. (2014). Does awareness effect the restorative function and perception of street trees?. Front. Psychol..

[123] Gathright J., Yamada Y., Morita M. (2006). Comparison of the physiological and psychological benefits of tree and tower climbing. Urban For. Urban Green..

[124] Morita E., Fukuda S., Nagano J., Hamajima N., Yamamoto H., Iwai Y., Nakashima T., Ohira H., Shirakawa T. (2007). Psychological effects of forest environments on healthy adults: Shinrin-yoku (forest-air bathing, walking) as a possible method of stress reduction. Public Health.

[125] Park B.-J., Furuya K., Kasetani T., Takayama N., Kagawa T., Miyazaki Y. (2011). Relationship between psychological responses and physical environments in forest settings. Landsc. Urban Plan..

[126] Shin Y.-K., Kim D.J., Jung-Choi K., Son Y., Koo J.-W., Min J.-A., Chae J.-H. (2013). Differences of psychological effects between meditative and athletic walking in a forest and gymnasium. Scand. J. For. Res..

[127] Joung D., Kim G., Choi Y., Lim H., Park S., Woo J.-M., Park B.-J. (2015). The prefrontal cortex activity and psychological effects of viewing forest landscapes in autumn season. Int. J. Environ. Res. Public Health.

[128] Kühn S., Düzel S., Eibich P., Krekel C., Wüstemann H., Kolbe J., Martensson J., Goebel J., Gallinat J., Wagner G.G. (2017). In search of features that constitute an “enriched environment” in humans: Associations between geographical properties and brain structure. Sci. Rep..

[129] Akpinar A., Barbosa-Leiker C., Brooks K.R. (2016). Does green space matter? Exploring relationships between green space type and health indicators. Urban For. Urban Green..

[130] Gilchrist K., Brown C., Montarzino A. (2015). Workplace settings and wellbeing: Greenspace use and views contribute to employee wellbeing at peri-urban business sites. Landsc. Urban Plan..

[131] Kim J.-H., Lee C., Sohn W. (2016). Urban natural environments, obesity, and health-related quality of life among Hispanic children living in inner-city neighborhoods. Int. J. Environ. Res. Public Health.

[132] Roe J., Aspinall P. (2011). The restorative outcomes of forest school and conventional school in young people with good and poor behaviour. Urban For. Urban Green..

[133] Gathright J., Yamada Y., Morita M. (2008). Tree-assisted therapy: Therapeutic and societal benefits from purpose-specific technical recreational tree-climbing programs. Arboric. Amp Urban For..

[134] An K.W., Kim E.I., Jeon K.S., Setsu T. (2004). Effects of forest stand density on human’s physiopsychological changes. J. Fac. Agric. Kyushu Univ..

[135] Wolf L.J., zu Ermgassen S., Balmford A., White M., Weinstein N. (2017). Is variety the spice of life? An experimental investigation into the effects of species richness on self-reported mental well-being. PLoS ONE.

[136] Martens D., Gutscher H., Bauer N. (2011). Walking in “wild” and “tended” urban forests: The impact on psychological well-being. J. Environ. Psychol..

[137] Park B.-J., Tsunetsugu Y., Kasetani T., Hirano H., Kagawa T., Sato M., Miyazaki Y. (2007). Physiological effects of shinrin-yoku (taking in the atmosphere of the forest)—Using salivary cortisol and cerebral activity as indicators. J. Physiol. Anthropol..

[138] Yamaguchi M., Deguchi M., Miyazaki Y. (2006). The effects of exercise in forest and urban environments on sympathetic nervous activity of normal young adults. J. Int. Med. Res..

[139] Tsunetsugu Y., Park B.-J., Ishii H., Hirano H., Kagawa T., Miyazaki Y. (2007). Physiological effects of shinrin-yoku (taking in the atmosphere of the forest) in an old-growth broadleaf forest in Yamagata Prefecture, Japan. J. Physiol. Anthropol..

[140] Lee J., Park B.-J., Tsunetsugu Y., Kagawa T., Miyazaki Y. (2009). Restorative effects of viewing real forest landscapes, based on a comparison with urban landscapes. Scand. J. For. Res..

[141] Lee J., Park B.-J., Tsunetsugu Y., Ohira T., Kagawa T., Miyazaki Y. (2011). Effect of forest bathing on physiological and psychological responses in young Japanese male subjects. Public Health.

[142] Park B.J., Tsunetsugu Y., Kasetani T., Kagawa T., Miyazaki Y. (2010). The physiological effects of Shinrin-yoku (taking in the forest atmosphere or forest bathing): Evidence from field experiments in 24 forests across Japan. Environ. Health Prev. Med..

[143] Tsunetsugu Y., Lee J., Park B.-J., Tyrväinen L., Kagawa T., Miyazaki Y. (2013). Physiological and psychological effects of viewing urban forest landscapes assessed by multiple measurements. Landsc. Urban Plan..

[144] Kobayashi H., Song C., Ikei H., Kagawa T., Miyazaki Y. (2015). Analysis of individual variations in autonomic responses to urban and forest environments. Evid. Based Complement. Alternat. Med..

[145] Hartig T., Evans G.W., Jamner L.D., Davis D.S., Gärling T. (2003). Tracking restoration in natural and urban field settings. J. Environ. Psychol..

[146] Yu C.-P., Lin C.-M., Tsai M.-J., Tsai Y.-C., Chen C.-Y. (2017). Effects of short forest bathing program on autonomic nervous system activity and mood states in middle-aged and elderly individuals. Int. J. Environ. Res. Public Health.

[147] Song C., Ikei H., Lee J., Park B.-J., Kagawa T., Miyazaki Y. (2013). Individual differences in the physiological effects of forest therapy based on Type A and Type B behavior patterns. J. Physiol. Anthropol..

[148] Song C., Ikei H., Kobayashi M., Miura T., Taue M., Kagawa T., Li Q., Kumeda S., Imai M., Miyazaki Y. (2015). Effect of forest walking on autonomic nervous system activity in middle-aged hypertensive individuals: A pilot study. Int. J. Environ. Res. Public Health.

[149] Horiuchi M., Endo J., Akatsuka S., Aaa A., Hasegawa T., Seko Y. (2013). Influence of forest walking on blood pressure, profile of mood states and stress markers from the viewpoint of aging. J. Aging Gerontol..

[150] Toda M., Den R., Hasegawa-Ohira M., Morimoto K. (2013). Effects of woodland walking on salivary stress markers cortisol and chromogranin A. Complement. Ther. Med..

[151] Horiuchi M., Endo J., Takayama N., Murase K., Nishiyama N., Saito H., Fujiwara A. (2014). Impact of viewing vs. not viewing a real forest on physiological and psychological responses in the same setting. Int. J. Environ. Res. Public Health.

[152] Beyer K., Kaltenbach A., Szabo A., Bogar S., Nieto F., Malecki K. (2014). Exposure to neighborhood green space and mental health: Evidence from the survey of the health of Wisconsin. Int. J. Environ. Res. Public Health.

[153] Egorov A.I., Griffin S.M., Converse R.R., Styles J.N., Sams E.A., Wilson A., Jackson L.E., Wade T.J. (2017). Vegetated land cover near residence is associated with reduced allostatic load and improved biomarkers of neuroendocrine, metabolic and immune functions. Environ. Res..

[154] Townsend J., Ilvento T., Barton S. (2016). Exploring the relationship between trees and human stress in the urban environment. Arboric. Urban For..

[155] Annerstedt M., Norman J., Boman M., Mattsson L., Grahn P., Währborg P. (2010). Finding stress relief in a forest. Ecol. Bull..

[156] Frumkin H., Bratman G.N., Breslow S.J., Cochran B., Kahn P.H., Lawler J.J., Levin P.S., Tandon P.S., Varanasi U., Wolf K.L. (2017). Nature contact and human health: A research agenda. Environ. Health Perspect..

[157] Jiang B., Chang C.-Y., Sullivan W.C. (2014). A dose of nature: Tree cover, stress reduction, and gender differences. Landsc. Urban Plan..

[158] Jiang B., Li D., Larsen L., Sullivan W.C. (2016). A dose-response curve describing the relationship between urban tree cover density and self-reported stress recovery. Environ. Behav..

[159] Tyrväinen L., Ojala A., Korpela K., Lanki T., Tsunetsugu Y., Kagawa T. (2014). The influence of urban green environments on stress relief measures: A field experiment. J. Environ. Psychol..

[160] Hauru K., Lehvävirta S., Korpela K., Kotze D.J. (2012). Closure of view to the urban matrix has positive effects on perceived restorativeness in urban forests in Helsinki, Finland. Landsc. Urban Plan..

[161] Kaplan R., Douglas I., Goode D., Houck M., Wang R. (2011). Intrinsic and aesthetic values of urban nature. Handbook of Urban Ecology.

[162] Van den Berg A.E., Jorgensen A., Wilson E.R. (2014). Evaluating restoration in urban green spaces: Does setting type make a difference?. Landsc. Urban Plan..

[163] Shin W.S. (2007). The influence of forest view through a window on job satisfaction and job stress. Scand. J. For. Res..

[164] Jung W.H., Woo J.-M., Ryu J.S. (2015). Effect of a forest therapy program and the forest environment on female workers’ stress. Urban For. Urban Green..

[165] Dolling A., Nilsson H., Lundell Y. (2017). Stress recovery in forest or handicraft environments—An intervention study. Urban For. Urban Green..

[166] Kim W., Lim S.-K., Chung E.-J., Woo J.-M. (2009). The effect of cognitive behavior therapy-based psychotherapy applied in a forest environment on physiological changes and remission of major depressive disorder. Psychiatry Investig..

[167] Berman M.G., Kross E., Krpan K.M., Askren M.K., Burson A., Deldin P.J., Kaplan S., Sherdell L., Gotlib I.H., Jonides J. (2012). Interacting with nature improves cognition and affect for individuals with depression. J. Affect. Disord..

[168] Sonntag-Öström E., Nordin M., Lundell Y., Dolling A., Wiklund U., Karlsson M., Carlberg B., Slunga Järvholm L. (2014). Restorative effects of visits to urban and forest environments in patients with exhaustion disorder. Urban For. Urban Green..

[169] Taylor M.S., Wheeler B.W., White M.P., Economou T., Osborne N.J. (2015). Urban street tree density and antidepressant prescription rates—A cross-sectional study in London, UK. Landsc. Urban Plan..

[170] Wu J., Jackson L. (2017). Inverse relationship between urban green space and childhood autism in California elementary school districts. Environ. Int..

[171] Van den Berg A.E., van den Berg C.G. (2011). A comparison of children with ADHD in a natural and built setting. Child Care Health Dev..

[172] Nordh H., Grahn P., Währborg P. (2009). Meaningful activities in the forest, a way back from exhaustion and long-term sick leave. Urban For. Urban Green..

[173] Ohtsuka Y., Yabunaka N., Takayama S. (1998). Shinrin-yoku (forest-air bathing and walking) effectively decreases blood glucose levels in diabetic patients. Int. J. Biometeorol..

[174] Ulrich R. (1984). View through a window may influence recovery from surgery. Science.

[175] Li Q., Kobayashi M., Kawada T. (2008). Relationships between percentage of forest coverage and standardized mortality ratios (SMR) of cancers in all prefectures in Japan. Open Public Health J..

[176] Abelt K., McLafferty S. (2017). Green streets: Urban green and birth outcomes. Int. J. Environ. Res. Public Health.

[177] Donovan G.H., Michael Y.L., Butry D.T., Sullivan A.D., Chase J.M. (2011). Urban trees and the risk of poor birth outcomes. Health Place.

[178] Cusack L., Larkin A., Carozza S.E., Hystad P. (2017). Associations between multiple green space measures and birth weight across two US cities. Health Place.

[179] Dadvand P., Ostro B., Figueras F., Foraster M., Basagaña X., Valentín A., Martinez D., Beelen R., Cirach M., Hoek G. (2014). Residential proximity to major roads and term low birth weight: The roles of air pollution, heat, noise, and road-adjacent trees. Epidemiology.

[180] Li Q., Morimoto K., Kobayashi M., Inagaki H., Katsumata M., Hirata Y., Hirata K., Shimizu T., Li Y.J., Wakayama Y. (2008). A forest bathing trip increases human natural killer activity and expression of anti-cancer proteins in female subjects. J. Biol. Regul. Homeost. Agents.

[181] Seo S.C., Park S.J., Park C.-W., Yoon W.S., Choung J.T., Yoo Y. (2015). Clinical and immunological effects of a forest trip in children with asthma and atopic dermatitis. Iran. J. Allergy Asthma Immunol..

[182] Li Q., Morimoto K., Kobayashi M., Inagaki H., Katsumata M., Hirata Y., Hirata K., Suzuki H., Li Y.J., Wakayama Y. (2008). Visiting a forest, but not a city, increases human natural killer activity and expression of anti-cancer proteins. Int. J. Immunopathol. Pharmacol..

[183] Li Q., Kobayashi M., Inagaki H., Hirata Y., Li Y.J., Hirata K., Shimizu T., Suzuki H., Katsumata M., Wakayama Y. (2010). A day trip to a forest park increases human natural killer activity and the expression of anti-cancer proteins in male subjects. J. Biol. Regul. Homeost. Agents.

[184] Li Q., Kobayashi M., Wakayama Y., Inagaki H., Katsumata M., Hirata Y., Hirata K., Shimizu T., Kawada T., Park B.J. (2009). Effect of phytoncide from trees on human natural killer cell function. Int. J. Immunopathol. Pharmacol..

[185] Mao G.X., Lan X.G., Cao Y.B., Chen Z.M., He Z.H., Lv Y.D., Wang Y.Z., Hu X.L., Wang G.F., Yan J. (2012). Effects of short-term forest bathing on human health in a broad-leaved evergreen forest in Zhejiang Province, China. Biomed. Environ. Sci..

[186] Larsen K., Gilliland J., Hess P., Tucker P., Irwin J., He M. (2009). The influence of the physical environment and sociodemographic characteristics on children’s mode of travel to and from school. Am. J. Public Health.

[187] Nehme E.K., Oluyomi A.O., Calise T.V., Kohl H.W. (2016). Environmental correlates of recreational walking in the neighborhood. Am. J. Health Promot..

[188] Taylor A.F., Wiley A., Kuo F.E., Sullivan W.C. (1998). Growing up in the inner city: Green spaces as places to grow. Environ. Behav..

[189] Janssen I., Rosu A. (2015). Undeveloped green space and free-time physical activity in 11 to 13-year-old children. Int. J. Behav. Nutr. Phys. Act..

[190] Eichinger M., Titze S., Haditsch B., Dorner T.E., Stronegger W.J. (2015). How are physical activity behaviors and cardiovascular risk factors associated with characteristics of the built and social residential environment?. PLoS ONE.

[191] Tilt J.H. (2010). Walking trips to parks: Exploring demographic, environmental factors, and preferences for adults with children in the household. Prev. Med..

[192] Zuniga-Teran A., Orr B., Gimblett R., Chalfoun N., Guertin D., Marsh S. (2017). Neighborhood design, physical activity, and wellbeing: Applying the Walkability Model. Int. J. Environ. Res. Public Health.

[193] Takano T., Nakamura K., Watanabe M. (2002). Urban residential environments and senior citizens’ longevity in megacity areas: The importance of walkable green spaces. J. Epidemiol. Community Health.

[194] Jones B.A. (2016). Work more and play less? Time use impacts of changing ecosystem services: The case of the invasive emerald ash borer. Ecol. Econ..

[195] Markevych I., Smith M.P., Jochner S., Standl M., Brüske I., von Berg A., Bauer C.-P., Fuks K., Koletzko S., Berdel D. (2016). Neighbourhood and physical activity in German adolescents: GINIplus and LISAplus. Environ. Res..

[196] Fan J.X., Wen M., Kowaleski-Jones L. (2014). An ecological analysis of environmental correlates of active commuting in urban U.S. Health Place.

[197] Wilson L.-A., Giles-Corti B., Turrell G. (2012). The association between objectively measured neighbourhood features and walking for transport in mid-aged adults. Local Environ..

[198] World Health Organization Body Mass Index—BMI. http://www.euro.who.int/en/health-topics/disease-prevention/nutrition/a-healthy-lifestyle/body-mass-index-bmi.

[199] Kim J.-H., Lee C., Olvera N.E., Ellis C.D. (2014). The role of landscape spatial patterns on obesity in Hispanic children residing in inner-city neighborhoods. J. Phys. Act. Health.

[200] Dadvand P., Villanueva C.M., Font-Ribera L., Martinez D., Basagaña X., Belmonte J., Vrijheid M., Gražulevičienė R., Kogevinas M., Nieuwenhuijsen M.J. (2014). Risks and benefits of green spaces for children: A cross-sectional study of associations with sedentary behavior, obesity, asthma, and allergy. Environ. Health Perspect..

[201] Lovasi G.S., Bader M.D.M., Quinn J., Neckerman K., Weiss C., Rundle A. (2012). Body mass index, safety hazards, and neighborhood attractiveness. Am. J. Prev. Med..

[202] Lovasi G.S., Schwartz-Soicher O., Neckerman K.M., Konty K., Kerker B., Quinn J., Rundle A. (2013). Aesthetic amenities and safety hazards associated with walking and bicycling for transportation in New York City. Ann. Behav. Med..

[203] Ulmer J.M., Wolf K.L., Backman D.R., Tretheway R.L., Blain C.J., O’Neil-Dunne J.P., Frank L.D. (2016). Multiple health benefits of urban tree canopy: The mounting evidence for a green prescription. Health Place.

[204] Lovasi G.S., Schwartz-Soicher O., Quinn J.W., Berger D.K., Neckerman K.M., Jaslow R., Lee K.K., Rundle A. (2013). Neighborhood safety and green space as predictors of obesity among preschool children from low-income families in New York City. Prev. Med..

[205] Ghimire R., Ferreira S., Green G.T., Poudyal N.C., Cordell H.K., Thapa J.R. (2017). Green space and adult obesity in the United States. Ecol. Econ..

[206] Tsai W.-L., Floyd M.F., Leung Y.-F., McHale M.R., Reich B.J. (2016). Urban vegetative cover fragmentation in the U.S. Am. J. Prev. Med..

[207] Lee J., Tsunetsugu Y., Takayama N., Park B.-J., Li Q., Song C., Komatsu M., Ikei H., Tyrväinen L., Kagawa T. (2014). Influence of forest therapy on cardiovascular relaxation in young adults. Evid. Based Complement. Alternat. Med..

[208] Li Q., Otsuka T., Kobayashi M., Wakayama Y., Inagaki H., Katsumata M., Hirata Y., Li Y., Hirata K., Shimizu T. (2011). Acute effects of walking in forest environments on cardiovascular and metabolic parameters. Eur. J. Appl. Physiol..

[209] Song C., Ikei H., Kobayashi M., Miura T., Li Q., Kagawa T., Kumeda S., Imai M., Miyazaki Y. (2017). Effects of viewing forest landscape on middle-aged hypertensive men. Urban For. Urban Green..

[210] Park B.-J., Tsunetsugu Y., Kasetani T., Morikawa T., Kagawa T., Miyazaki Y. (2009). Physiological effects of forest recreation in a young conifer forest in Hinokage Town, Japan. Silva Fenn..

[211] Mao G.-X., Cao Y.-B., Lan X.-G., He Z.-H., Chen Z.-M., Wang Y.-Z., Hu X.-L., Lv Y.-D., Wang G.-F., Yan J. (2012). Therapeutic effect of forest bathing on human hypertension in the elderly. J. Cardiol..

[212] Sung J., Woo J.-M., Kim W., Lim S.-K., Chung E.-J. (2012). The effect of cognitive behavior therapy-based “forest therapy” program on blood pressure, salivary cortisol level, and quality of life in elderly hypertensive patients. Clin. Exp. Hypertens..

[213] Donovan G.H., Butry D.T., Michael Y.L., Prestemon J.P., Liebhold A.M., Gatziolis D., Mao M.Y. (2013). The relationship between trees and human health. Am. J. Prev. Med..

[214] Wu J., Rappazzo K.M., Simpson R.J., Joodi G., Pursell I.W., Mounsey J.P., Cascio W.E., Jackson L.E. (2018). Exploring links between greenspace and sudden unexpected death: A spatial analysis. Environ. Int..

[215] Kardan O., Gozdyra P., Misic B., Moola F., Palmer L.J., Paus T., Berman M.G. (2015). Neighborhood greenspace and health in a large urban center. Sci. Rep..

[216] Donovan G.H., Michael Y.L., Gatziolis D., Prestemon J.P., Whitsel E.A. (2015). Is tree loss associated with cardiovascular-disease risk in the Women’s Health Initiative? A natural experiment. Health Place.

[217] Tarar G., Etheredge C.L., McFarland A., Snelgrove A., Waliczek T.M., Zajicek J.M. (2015). The effect of urban tree canopy cover and vegetation levels on incidence of stress-related illnesses in humans in metropolitan statistical areas of Texas. HortTechnology.

[218] Kim E.S., Kawachi I. (2017). Perceived neighborhood social cohesion and preventive healthcare use. Am. J. Prev. Med..

[219] Sullivan W.C., Kuo F.E., Depooter S.F. (2004). The fruit of urban nature: Vital neighborhood spaces. Environ. Behav..

[220] Holtan M.T., Dieterlen S.L., Sullivan W.C. (2015). Social life under cover: Tree canopy and social capital in Baltimore, Maryland. Environ. Behav..

[221] Piff P.K., Dietze P., Feinberg M., Stancato D.M., Keltner D. (2015). Awe, the small self, and prosocial behavior. J. Pers. Soc. Psychol..

[222] Twohig-Bennett C., Jones A. (2018). The health benefits of the great outdoors: A systematic review and meta-analysis of greenspace exposure and health outcomes. Environ. Res..

[223] Zeng Y., Dong L. (2015). Thermal human biometeorological conditions and subjective thermal sensation in pedestrian streets in Chengdu, China. Int. J. Biometeorol..

[224] Hägerhäll C.M., Ode A., Tveit M.S., Velarde M.D., Colfer C.J.P., Sarjala T., Mery G., Katila P., Galloway G., Alfaro R.I., Kanninen M., Lobovikov M., Varjo J. (2010). Forests, human health and well-being in light of climate change and urbanization. Forests and Society: Responding to Global Drivers of Change.

[225] Romanelli C., Capon A., Maiero M., Campbell-Lendrum D., Romanelli C., Cooper D., Campbell-Lendrum D., Maiero M., Karesh W.B., Hunter D., Golden C.D. (2015). Climate change, biodiversity and human health. Connecting Global Priorities: Biodiversity and Human Health: A State Of Knowledge Review.

[226] Watts N., Adger W.N., Agnolucci P., Blackstock J., Byass P., Cai W., Chaytor S., Colbourn T., Collins M., Cooper A. (2015). Health and climate change: Policy responses to protect public health. Lancet.

[227] Watts N., Amann M., Arnell N., Ayeb-Karlsson S., Belesova K., Berry H., Bouley T., Boykoff M., Byass P., Cai W. (2018). The 2018 report of the Lancet Countdown on health and climate change: Shaping the health of nations for centuries to come. Lancet.

[228] Barron S., Nitoslawski S., Wolf K.L., Woo A., Desautels E., Sheppard S.R.J. (2019). Greening blocks: A conceptual typology of practical design interventions to integrate health and climate resilience co-benefits. Int. J. Environ. Res. Public Health.

[229] Romanelli C., Cooper D., Campbell-Lendrum D., Maiero M., Karesh W.B., Hunter D., Golden C.D. (2015). Connecting Global Priorities: Biodiversity and Human Health: A State Of Knowledge Review.

[230] McDonald R., Aljabar L., Aubuchon C., Birnbaum H.G., Chandler C., Toomey B., Daley J., Jimenez W., Trieschman E., Paque J. (2017). Funding Trees For Health: An Analysis of Finance and Policy Actions To Enable Tree Planting For Public Health.

[231] Sanusi R., Johnstone D., May P., Livesley S.J. (2016). Street orientation and side of the street greatly influence the microclimatic benefits street trees can provide in summer. J. Environ. Qual..

[232] Lin T.-P., Matzarakis A., Hwang R.-L. (2010). Shading effect on long-term outdoor thermal comfort. Build. Environ..

[233] Pearson A., Jordan Z., Munn Z. (2012). Translational science and evidence-based healthcare: A clarification and reconceptualization of how knowledge is generated and used in healthcare. Nurs. Res. Pract..

[234] Wolf K.L. (2018). Cascading Benefits: Designing Green Stormwater Infrastructure for Human Wellness.

[235] Shanahan D.F., Lin B.B., Bush R., Gaston K.J., Dean J.H., Barber E., Fuller R.A. (2015). Toward improved public health outcomes from urban nature. Am. J. Public Health.

[236] Jennings V., Gaither C. (2015). Approaching environmental health disparities and green spaces: An ecosystem services perspective. Int. J. Environ. Res. Public Health.

[237] Wolf K.L., Ferrini F., Konijnendijk van den Bosch C.C., Fini A. (2017). Social aspects of urban forestry and metro nature. Routledge Handbook of Urban Forestry.

