# Supplementary material for: Urban Trees and Human Health: A Scoping Review
Source: Int J Environ Res Public Health. 2020 Jun 18;17(12):4371. doi: 10.3390/ijerph17124371 (PMC7345658; doi:10.3390/ijerph17124371)
Supplement: Supplementary file 1 [file ijerph-17-04371-s001.pdf]

Supplementary Material

Urban trees and human health: A scoping review

Authors: Kathleen L. Wolf (corresponding author – kwolf@uw.edu), Sharon T. Lam, Jennifer K. McKeen, Gregory R.A. Richardson, Matilda van den Bosch, Adrina C. Bardekjian

Table S1: Citations and References for Table 2

All studies included in scoping review are listed by the domains and subdomains of the health outcomes conceptual framework, then sorted by study design. This table corresponds to Table 2 in the manuscript. All 201 studies (in 199 articles) are cited in table, and references follow.

Doman/Subdomain	Study Count	Experiment	Natural/ Quasi-Experiment	Longitudinal/ Cohort	Cross Sectional	Modelling	Time Series
**4.1. REDUCING HARM**	**82**	.	.	.	.	.	.
**4.1.1 Air Pollutants and Respiratory Condition**	14	.	.	.	Lovasi et al. 2008; Wang et al. 2016; Akerman et al. 2003*; Pilat et al. 2012*; Alcock et al. 2017*; Lovasi et al. 2013a**	Tiwary et al. 2009; Nowak et al. 2013, 2014; Donovan et al. 2016; Hirabayashi & Nowak 2016; Rao et al. 2014, 2017; Ratola et al. 2017	.
**4.1.2 Tree Pollen and VOCs**	40	Song et al. 2016b*	Loureiro 2005*	Cebrino et al. 2017**; Irani et al. 2013**; Weichenthal et al. 2016**	Ishizaki et al. 1987*; Guerra et al. 1995*; Vieira et al. 1998*; Sam et al. 1998*; Harmanci & Mahesh et al. 2010*; Metintas 2000*; Lin et al. 2002*; Armentia et al. 2004*; Sánchez-Mesa et al. 2005*; Fuhrman et al. 2007*; Li et al. 2009*; Sheehan et al. 2010*; Puiggròs et al. 2015*; Sung et al. 2017*; Alcázar et al. 2004**; Diaz de la Guardia et al. 2006**; Motreff et al. 2014**; Kim et al. 2016a**	Cariñanos et al. 2016**, 2017**; Ren et al. 2017**	Celik et al. 2005*; Gonianakis et al. 2006*; Darrow et al. 2012*; Osborne et al. 2017*; Dales et al. 2004**, 2008**; Diaz et al. 2007**; May et al. 2011**; Sheffield et al. 2011**; Cakmak et al. 2012**; Jariwala et al. 2011**, 2014**; Caillaud et al. 2015**; Jeon-Slaughter et al. 2016**
**4.1.3 Ultraviolet Radiation**	5	.	Boldemann et al. 2004, 2006	.	.	Heisler et al. 2003; Parisi et al. 2000a*, 2000b*	.

Doman/Subdomain	Study Count	Experiment	Natural/ Quasi-Experiment	Longitudinal/ Cohort	Cross Sectional	Modelling	Time Series
**4.1.4 Excess Heat and Thermal Comfort**	17		Lin et al. 2012, 2013; Zeng & Dong 2015; Jeong et al. 2016; Song & Jeong 2016	Kilbourne et al. 1982; Graham et al. 2016	.	Johansson & Emmanuel 2006; Stone et al. 2014; de Abreu-Harbich et al. 2015; Klemm et al. 2015; Lee et al. 2016; Sanusi et al. 2016; Wu et al. 2016; Milošević et al. 2017; Kántor et al. 2018; Lin et al. 2010*	
**4.1.5 Crime**	6	.	Kuo & Sullivan 2001b; Kondo et al. 2017a*; Kondo et al. 2017b		Troy et al. 2012; Gilstad-Hayden et al. 2015; Donovan & Prestemon 2012*	.	.
**4.2. RESTORING CAPACITIES**	**63**	**.**	**.**	**.**	**.**	**.**	**.**
**4.2.1 Cognition and Attention Restoration**	13	Gathright et al. 2006 (second study); Berman et al. 2008; Shin et al. 2011; Martinez-Soto et al. 2013; Lin et al. 2014; Takayama et al. 2014; Perkins, et al. 2011*; Gatersleben, et al. 2013*	Kuo 2001; Kuo & Sullivan 2001a; Paddle et al. 2016; Taylor et al. 2002*		Mårtensson et al. 2009	.	.
**4.2.2 Mental Health, Anxiety and Mood**	15	An et al. 2004; Gathright et al. 2006; Morita et al. 2007; Park et al. 2011; Roe & Aspinall 2011; Shin et al. 2013; Van den Berg et al. 2014; Joung et al. 2015; Wolf et al. 2017; Gathright et al. 2008*; Martens et al. 2011*	Gilchrist et al. 2015	.	Kühn et al. 2017; Akpinar et al. 2016*; Reid et al. 2017*	.	.

Doman/Subdomain	Study Count	Experiment	Natural/ Quasi-Experiment	Longitudinal/ Cohort	Cross Sectional	Modelling	Time Series
**4.2.3 Psychophysiological Stress**	25	Hartig et al. 2003; Yamaguchi et al. 2006; Park et al. 2007, 2010; Lee et al. 2009, 2011; Tsunetsugu et al. 2007, 2013; Gatersleben & Andrews 2013 (second study); Horiuchi et al. 2013; Tyrväinen et al. 2014; Kobayashi et al. 2015; Jiang et al. 2016; Yu et al. 2017; Toda et al. 2013*; Horiuchi et al. 2014*; Jiang et al. 2014*; Jung et al. 2015*	Shin 2007; Hauru et al. 2012	Dolling et al. 2017*	Annerstedt et al. 2010; Beyer et al. 2014; Townsend et al. 2016; Egorov et al. 2017	.	.
**4.2.4 Clinical Outcomes**	10	Kim et al. 2009; Berman et al. 2012; Sonntag-Öström et al. 2014; van den Berg & van den Berg 2011*	Nordh et al. 2009*	Ulrich 1984; Ohtsuka et al. 1998	Li et al. 2008a; Taylor et al. 2015; Wu & Jackson 2017	.	.
**4.3. BUILDING CAPACITIES**	**56**	**.**	**.**	**.**	**.**	**.**	**.**
**4.3.1 Birth Outcomes**	4	.	.	Donovan et al. 2011*; Dadvand et al. 2014a*; Cusack et al. 2017*	Abelt & McLafferty 2017	.	.
**4.3.2 Immune System**	**6**	Li et al. 2007, 2008b, 2008c, 2010; Mao et al. 2012b; Seo et al. 2014	.	.	.	.	.

Doman/Subdomain	Study Count	Experiment	Natural/ Quasi-Experiment	Longitudinal/ Cohort	Cross Sectional	Modelling	Time Series
**4.3.3 Active Living**	19	.	Fjørtoft 2001; Jones 2016	.	Taylor et al. 1998; Takano et al. 2002; Larsen et al. 2009; Tilt, 2010; Lovasi et al. 2011; Nehme et al. 2013; Schipperijn et al. 2013; Eichinger et al. 2015; Janssen & Rosu 2015; Zuniga-Teran, 2017; Lovasi et al. 2013b*; Compernolle et al. 2016*; Costigan et al. 2017*; Markevych et al. 2016*; Mertens et al. 2017*; Wilson et al. 2011**; Fan et al. 2014**		.
**4.3.4 Weight Status**	8	.	.	.	Lovasi et al. 2012, 2013c; Dadvand et al. 2014b; Kim, et al. 2014, 2016b; Tsai et al. 2016; Ulmer et al. 2016; Ghimire et al. 2017	.	.
**4.3.5 Cardiovascular Function**	16	Park et al. 2009; Li et al. 2011; Matsunaga et al. 2011; Mao et al. 2012a; Lee et al. 2014; Song et al. 2015a, 2017a; Sung et al. 2012*; Song et al. 2013*	Donovan et al. 2013, 2015; Song et al. 2017b; Song et al. 2015b*	.	Kardan et al. 2015; Tarar et al. 2015*; Wu et al. 2018*	.	.
**4.3.6 Social Cohesion**	3	.	Sullivan et al. 2004	.	Holtan et al. 2015; Piff et al. 2015	.	.
**TOTAL STUDIES**	**201**	**57**	**26**	**12**	**69**	**24**	**13**

Notes: *indicates mixed or neutral findings. ** indicates negative findings.

References

Abelt K, McLafferty S. 2017. Green streets: Urban green and birth outcomes. Int J Environ Res Public Health 14(7):771, https://doi.org/10.3390/ijerph14070771.

Akerman M, Valentine-Maher S, Rao M, Taningco G, Khan R, Tuysugoglu G, Joks R. 2003. Allergen sensitivity and asthma severity at an inner city asthma center. J Asthma 40(1):55-62, https://doi.org/10.1081/JAS-120017207.

Akpinar A, Barbosa-Leiker C, Brooks KR. 2016. Does green space matter? Exploring relationships between green space type and health indicators. Urban For Urban Gree 20:407-418, https://doi.org/10.1016/j.ufug.2016.10.013.

Alcázar P, Cariñanos P, De Castro C, Guerra F, Moreno C, Dominguez-Vilches E, Galán C. 2004. Airborne plane-tree (*Platanus hispanica*) pollen distribution in the city of Córdoba, southwestern Spain, and possible implications on pollen allergy. J Investig Allergol Clin Immunol 14(3):238-243.

Alcock I, White M, Cherrie M, Wheeler B, Taylor J, McInnes R, Otte Im Kampe E, Vardoulakis S, Sarran C, et al. 2017. Land cover and air pollution are associated with asthma hospitalisations: A cross-sectional study. Environ Int 109:29-41, https://doi.org/10.1016/j.envint.2017.08.009.

An KW, Kim EI, Joen KS, Setsu T. 2004. Effects of forest stand density on human's physiopsychological changes. J Faculty of Agriculture, Kyushu Univ 49(2):283-291.

Annerstedt M, Norman J, Boman M, Mattsson L, Grahn P, Währborg P. 2010. Finding stress relief in a forest. Ecological Bulletins 53:33-42.

Armentia A, Asensio T, Subiza J, Arranz ML, Gil F-M, Callejo A. 2004. Living in towers as risk factor of pollen allergy. Allergy 59(3):302-305.

Berman MG, Jonides J, Kaplan S. 2008. The cognitive benefits of interacting with nature. Psychol Sci 19(12):1207-1212, https://doi.org/10.1111/j.1467-9280.2008.02225.

Berman MG, Kross E, Krpan KM, Askren MK, Burson A, Deldin PJ, Kaplan S, Sherdell L, Gotlib IH, Jonides J. 2012. Interacting with nature improves cognition and affect for individuals with depression. J Affect Disord 140(3):300-305, https://doi.org/10.1016/j.jad.2012.03.012.

Beyer KM, Kaltenbach A, Szabo A, Bogar S, Nieto FJ, Malecki KM. 2014. Exposure to neighbourhood green space and mental health: Evidence from the Survey of the Health of Wisconsin. Int J Environ Res Public Health 11(3):3453-3472, https://doi.org/10.3390/ijerph110303453.

Boldemann C, Blennow M, Dal H, Mårtensson F, Raustorp A, Yuen K, Wester U. 2006. Impact of preschool environment upon children's physical activity and sun exposure. Prev Med 42(4):301-308, https://doi.org/10.1016/j.ypmed.2005.12.006.

Boldemann C, Dal H, Wester U. 2004. Swedish pre-school children's UVR exposure--a comparison between two outdoor environments. Photodermatol Photoimmunol Photomed 20(1):2-8, https://doi.org/10.1111/j.1600-0781.2004.00069.x.

Caillaud DM, Martin S, Ségala C, Vidal P, Lecadet J, Pellier S, Rouzaire P, Tridon A, Evrard B. 2015. Airborne pollen levels and drug consumption for seasonal allergic rhinoconjunctivitis: A 10-year study in France. Allergy 70(1):99-106, https://doi.org/10.1111/all.12522.

Cakmak S, Dales RE, Coates F. 2012. Does air pollution increase the effect of aeroallergens on hospitalization for asthma? J Allergy Clin Immunol 129(1):228-231, https://doi.org/10.1016/j.jaci.2011.09.025.

Cariñanos P, Adinolfi C, Díaz de la Guardia C, De Linares C, Casares-Porcel M. 2016. Characterization of allergen emission sources in urban areas. J Environ Qual 45:244-252, https://doi.org/10.2134/jeq2015.02.0075.

Cariñanos P, Casares-Porcel M, Díaz de la Guardia C, Aira MJ, Belmonte J, Boi M, Elvira-Rendueles B, De Linares C, Fernández-Rodriguez S, et al.al. 2017. Assessing allergenicity in urban parks: A nature-based solution to reduce the impact on public health. Environ Res 155:219-227, https:doi.//org/10.1016/j.envres.2017.02.015.

Cebrino J, Barasona MJ, Alcázar P, Moreno C, Domínguez-Vilches E, Galán C. 2017. Airborne pollen in Córdoba city (Spain) and its implications for pollen allergy. Aerobiologia 33(2):281-291, https://doi.org/10.1007/s10453-016-9469-8.

Celik G, Mungan D, Pinar M, Misirligil Z. 2005. Poplar pollen-related allergy in Ankara, Turkey: How important for patients living in a city with high pollen load? Allergy Asthma Proc 26(2):113-9.

Compernolle S, De Cocker K, Roda C, Oppert JM, Mackenbach JD, Lakerveld J, Glonti K, Bardos H, Rutter H, et al. 2016. Physical environmental correlates of domain-specific sedentary behaviours across five European regions (the SPOTLIGHT Project). PLoS One 11(10):e0164812, https://doi.org/10.1371/journal.pone.0164812.

Costigan SA, Veitch J, Crawford D, Carver A, Timperio A. 2017. A cross-sectional investigation of the importance of park features for promoting regular physical activity in parks. Int J Environ Res Public Health 14(11): 1335, https://doi.org/10.3390/ijerph14111335.

Cusack L, Larkin A, Carozza SE, Hystad P. 2017. Associations between multiple green space measures and birth weight across two US cities. Health Place 47:36-43, https://doi.org/10.1016/j.healthplace.2017.07.002.

Dadvand P, Ostro B, Figueras F, Foraster M, Basagaña X, Valentín A, Martinez D, Beelen R, Cirach M, Hoek G. 2014a. Residential proximity to major roads and term low birth weight: The roles of air pollution, heat, noise, and road-adjacent trees. Epidemiology 25(4):518-525 , https://doi.org/10.1097/EDE.0000000000000107.

Dadvand P, Villanueva CM, Font-Ribera L, Martinez D, Basagaña X, Belmonte J, Vrijheid M, Gražulevičienė R, Kogevinas M, Nieuwenhuijsen MJ. 2014b. Risks and benefits of green spaces for children: A cross-sectional study of associations with sedentary behavior, obesity, asthma, and allergy. Environ Health Perspect 122(12):1329-1335, https://doi.org/10.1289/ehp.1308038.

Dales RE, Cakmak S, Judek S, Coates F. 2008. Tree pollen and hospitalization for asthma in urban Canada. Int Arch Allergy Immunol 146(3):241-247, https://doi.org/10.1159/000116360.

Dales RE, Cakmak S, Judek S, Dann T, Coates F, Brook JR, Burnett RT. 2004. Influence of outdoor aeroallergens on hospitalization for asthma in Canada. J Allergy Clin Immunol 113(2):303-306, https://doi.org/10.1016/j.jaci.2003.11.016.

Darrow LA, Hess J, Rogers CA, Tolbert PE, Klein M, Sarnat SE. 2012. Ambient pollen concentrations and emergency department visits for asthma and wheeze. J Allergy Clin Immunol 130(3):630-638.e4, https://doi.org/10.1016/j.jaci.2012.06.020.

de Abreu-Harbich LV, Labaki LC, Matzarakis A. 2015. Effect of tree planting design and tree species on human thermal comfort in the tropics. Landsc Urban Plan 138:99-109, https://doi.org/10.1016/j.landurbplan.2015.02.008.

Diaz de la Guardia C, Alba F, de Linares C, Nieto-Lugilde D, López Caballero J. 2006. Aerobiological and allergenic analysis of Cupressaceae pollen in Granada (southern Spain). J Investig Allergol Clin Immunol 16(1):24-33.

Díaz J, Linares C, Tobâias A. 2007. Short-term effects of pollen species on hospital admissions in the city of Madrid in terms of specific causes and age. Aerobiologia 23(4):231-238, https://doi.org/10.1007/s10453-007-9067-x.

Dolling A, Nilsson H, Lundell Y. 2017. Stress recovery in forest or handicraft environments--an intervention study. Urban Forestry & Urban Greening 27:162-172 Urban For Urban Gree 27:162-172, https://doi.org/10.1016/j.ufug.2017.07.006.

Donovan GH, Butry DT, Michael YL, Prestemon JP, Liebhold AM, Gatziolis D, Mao MY. 2013. The relationship between trees and human health: Evidence from the spread of the emerald ash borer. Am J Prev Med 44(2):139-145, https://doi.org/10.1016/j.amepre.2012.09.066.

Donovan GH, Jovan SE, Gatziolis D, Burstyn I, Michael YL, Amacher MC, Monleon VJ. 2016. Using an epiphytic moss to identify previously unknown sources of atmospheric cadmium pollution. Sci Total Environ 559:84-93, https://doi.org/10.1016/j.scitotenv.2016.03.182.

Donovan GH, Michael YL, Butry DT, Sullivan AD, Chase JM. 2011. Urban trees and the risk of poor birth outcomes. Health Place 17(1):390-393, https://doi.org/10.1016/j.healthplace.2010.11.004.

Donovan GH, Michael YL, Gatziolis D, Prestemon JP, Whitsel EA. 2015. Is tree loss associated with cardiovascular-disease risk in the women's health initiative? A natural experiment. Health Place 36:1-7, https://doi.org/10.1016/j.healthplace.2015.08.007.

Donovan GH, Prestemon JP. 2012. The effect of trees on crime in Portland, Oregon. Environ Behav44(1):3-30, https://doi.org/10.1177/0013916510383238.

Egorov AI, Griffin SM, Converse RR, Styles JN, Sams EA, Wilson A, Jackson LE, Wade TJ. 2017. Vegetated land cover near residence is associated with reduced allostatic load and improved biomarkers of neuroendocrine, metabolic and immune functions. Environ Res 158:508-521, https://doi.org/10.1016/j.envres.2017.07.009.

Eichinger M, Titze S, Haditsch B, Dorner TE, Stronegger WJ. 2015. How are physical activity behaviors and cardiovascular risk factors associated with characteristics of the built and social residential environment? PLoS One 10(6):e0126010, https://doi.org/10.1371/journal.pone.0126010.

Fan JX, Wen M, Kowaleski-Jones L. 2014. An ecological analysis of environmental correlates of active commuting in urban U.S. Health Place 30:242-250, https://doi.org/10.1016/j.healthplace.2014.09.014.

Fjørtoft I. 2001. The natural environment as a playground for children: The impact of outdoor play activities in pre-primary school children. Early Child Educ J 29(2):111-117, https://doi.org/10.1023/A:1012576913074.

Fuhrman C, Sarter H, Thibaudon M, Delmas MC, Zeghnoun A, Lecadet J, Caillaud D. 2007. Short-term effect of pollen exposure on antiallergic drug consumption. Ann Allergy Asthma Immunol 99(3):225-231, https://doi.org/10.1016/S1081-1206(10)60657-6.

Gatersleben B, Andrews M. 2013. When walking in nature is not restorative - the role of prospect and refuge. Health Place 20:91-101, https://doi.org/doi:10.1016/j.healthplace.2013.01.001.

Gathright J, Yamada Y, Morita M. 2006. Comparison of the physiological and psychological benefits of tree and tower climbing. Urban For Urban Gree 5(3):141-149, https://doi.org/10.1016/j.ufug.2005.12.003.

Gathright J, Yamada Y, Morita M. 2008. Tree-assisted therapy: Therapeutic and societal benefits from purpose-specific technical recreational tree-climbing programs. Arboric Urban For 34(4):222-229.

Ghimire R, Ferreira S, Green GT, Poudyal NC, Cordell HK, Thapa JR. 2017. Green space and adult obesity in the United States. Ecol Econ 136:201-212, https://doi.org/10.1016/j.ecolecon.2017.02.002.

Gilchrist K, Brown C, Montarzino A. 2015. Workplace settings and wellbeing: Greenspace use and views contribute to employee wellbeing at peri-urban business sites. Landsc Urban Plan 138:32-40, https://doi.org/10.1016/j.landurbplan.2015.02.004.

Gilstad-Hayden K, Wallace LR, Carroll-Scott A, Meyer SR, Barbo S, Murphy-Dunning C, Ickovics JR. 2015. Greater tree canopy cover is associated with lower rates of both violent and property crime in New Haven, CT. Landsc Urban Plan 143:248-253, https://doi.org/10.1016/j.landurbplan.2015.08.005.

Gonianakis MI, Baritaki MA, Neonakis IK, Gonianakis IM, Kypriotakis Z, Darivianaki E, Bouros D, Kontou-Filli K. 2006. A 10-year aerobiological study (1994-2003) in the Mediterranean island of Crete, Greece: Trees, aerobiologic data, and botanical and clinical correlations. Allergy Asthma Proc 27(5):371-377, http://doi.org/10.2500/aap.2006.27.2911.

Graham DA, Vanos JK, Kenny NA, Brown RD. 2016. The relationship between neighbourhood tree canopy cover and heat-related ambulance calls during extreme heat events in Toronto, Canada. Urban For Urban Gree 20:180-186, https://doi.org/10.1016/j.ufug.2016.08.005.

Guerra F, Galán Carmen C, Daza JC, Miguel R, Moreno C, González J, Domínguez E. 1995. Study of sensitivity to the pollen of Fraxinus spp. (Oleaceae) in Cordoba, Spain. J Investig Allergol Clin Immunol 5(3):166-70.

Harmanci E, Metintas E. 2000. The type of sensitization to pollens in allergic patients in Eskisehir (Anatolia), Turkey. Allergol Immunopathol (Madr) 28(2):63-66.

Hartig T, Evans GW, Jamner LD, Davis DS, Gärling T. 2003. Tracking restoration in natural and urban field settings. J Environ Psychol 23(2):109-123, https://doi.org/10.1016/S0272-4944(02)00109-3.

Hauru K, Lehvävirta S, Korpela K, Kotze DJ. 2012. Closure of view to the urban matrix has positive effects on perceived restorativeness in urban forests in Helsinki, Finland. Landsc Urban Plan 107:361-369, https://doi.org/10.1016/j.landurbplan.2012.07.002.

Heisler GM, Grant RH, Gao W. 2003. Individual-and scattered-tree influences on ultraviolet irradiance. Agric For Meteorol 120(1-4):113-126, https://doi.org/10.1016/j.agrformet.2003.08.024.

Hirabayashi S, Nowak DJ. 2016. Comprehensive national database of tree effects on air quality and human health in the United States. Environ Pollut 215:48-57, https://doi.org/10.1016/j.envpol.2016.04.068.

Holtan MT, Dieterlen SL, Sullivan WC. 2015. Social life under cover: Tree canopy and social capital in Baltimore, Maryland. Environ Behav 47(5):502-525, https://doi.org/10.1177%2F0013916513518064.

Horiuchi M, Endo J, Akatsuka S, Uno T, Hasegawa T, Seko Y. 2013. Influence of forest walking on blood pressure, profile of mood states, and stress markers from the viewpoint of aging. J Aging Gerontol 1:9-17.

Horiuchi M, Endo J, Takayama N, Murase K, Nishiyama N, Saito H, Fujiwara A. 2014. Impact of viewing vs. not viewing a real forest on physiological and psychological responses in the same setting. Int J Environ Res Public Health 11(10):10883-10901, https://doi.org/10.3390/ijerph111010883.

Irani I, Karam K, Baz B, Maatouk M, Zaitoun Z. 2013. Airborne pollen concentrations and the incidence of allergic asthma and rhinoconjunctivitis in Lebanon. Revue Francaise d'Allergologie 53(5):441-445, https://doi.org/10.1016/j.reval.2012.12.002.

Ishizaki T, Koizumi K, Ikemori R, Ishiyama Y, Kushibiki E. 1987. Studies of prevalence of Japanese cedar pollinosis among the residents in a densely cultivated area. Ann Allergy 58(4):265-70.

Janssen I, Rosu A. 2015. Undeveloped green space and free-time physical activity in 11 to 13-year-old children. Int J Behav Nutr Phys Act 12(26):7, https://doi.org/10.1186/s12966-015-0187-3.

Jariwala S, Toh J, Shum M, de Vos G, Zou K, Sindher S, Patel P, Geevarghese A, Tavdy A, Rosenstreich D. 2014. The association between asthma-related emergency department visits and pollen and mold spore concentrations in the Bronx, 2001-2008. J Asthma 51(1):79-83, https://doi.org/10.3109/02770903.2013.853779.

Jariwala SP, Kurada S, Moday H, Thanjan A, Bastone L, Khananashvili M, Fodeman J, Hudes G, Rosenstreich D. 2011. Association between tree pollen counts and asthma ED visits in a high-density urban center. J Asthma 48(5):442-448, https://doi.org/10.3109/02770903.2011.567427.

Jeon-Slaughter H, Claassen CA, Khan DA, Mihalakos P, Lee KB, Brown ES. 2016. Temporal association between nonfatal self-directed violence and tree and grass pollen counts. J Clin Psychiatry 77(9):1160-1167, https://doi.org/10.4088/JCP.15m09864.

Jeong M, Park S, Song G. 2016. Comparison of human thermal responses between the urban forest area and the central building district in Seoul, Korea. Urban For Urban Gree 15:133-148, https://doi.org/10.1016/j.ufug.2015.12.005.

Jiang B, Chang C-Y, Sullivan WC. 2014. A dose of nature: Tree cover, stress reduction, and gender differences. Landsc Urban Plan 132:26-36, https://doi.org/10.1016/j.landurbplan.2014.08.005.

Jiang B, Li D, Larsen L, Sullivan WC. 2016. A dose-response curve describing the relationship between urban tree cover density and self-reported stress recovery. Environ Behav 48(4):607-629, https://doi.org/10.1177/0013916514552321.

Johansson E, Emmanuel R. 2006. The influence of urban design on outdoor thermal comfort in the hot, humid city of Colombo, Sri Lanka. Int J Biometeorol 51(2):119-133, https://doi.org/10.1007/s00484-006-0047-6.

Jones BA. 2016. Work more and play less? Time use impacts of changing ecosystem services: The case of the invasive emerald ash borer. Ecol Econ 124:49-58, https://doi.org/10.1016/j.ecolecon.2016.02.003.

Joung D, Kim G, Choi Y, Lim H, Park S, Woo J-M, Park B-J. 2015. The prefrontal cortex activity and psychological effects of viewing forest landscapes in autumn season. Int J Environ Res Public Health 12(7):7235-7243, https://doi.org/10.3390/ijerph120707235.

Jung WH, Woo J, Ryu JS. 2015. Effect of a forest therapy program and the forest environment on female workers' stress. Urban For Urban Gree 14(2):274-281, https://doi.org/10.1016/j.ufug.2015.02.004.

Kántor N, Chen L, Gál CV. 2018. Human-biometeorological significance of shading in urban public spaces - summertime measurements in Pécs, Hungary. Landsc Urban Plan 170:241-255, https://doi.org/10.1016/j.landurbplan.2017.09.030.

Kardan O, Gozdyra P, Misic B, Moola F, Palmer LJ, Paus T, Berman MG. 2015. Neighbourhood greenspace and health in a large urban center. Sci Rep 5:11610, https://doi.org/10.1038/srep11610.

Kilbourne EM, Choi K, Jones S. 1982. Risk factors for heatstroke. A case-control study. JAMA 247:3332-3336, https:doi.org/10.1001/jama.1982.03320490030031.

Kim DH, Park YS, Jang HJ, Kim JH, Lim DH. 2016a. Prevalence and allergen of allergic rhinitis in Korean children. Am J Rhinol Allergy 30(3):72-78, https://doi.org/10.2500/ajra.2013.27.4317.

Kim J-H, Lee C, Olvara NE, Ellis CD. 2014. The role of landscape spatial patterns on obesity in Hispanic children residing in inner-city neighbourhoods. J Phys Act Health 11:1449-1457, https://doi.org/10.1123/jpah.2012-0503.

Kim JH, Lee C, Sohn W. 2016b. Urban natural environments, obesity, and health-related quality of life among Hispanic children living in inner-city neighbourhoods. Int J Environ Res Public Health 13(1), https://doi.org/10.3390/ijerph13010121.

Kim W, Lim SK, Chung EJ, Woo JM. 2009. The effect of cognitive behavior therapy-based psychotherapy applied in a forest environment on physiological changes and remission of major depressive disorder. Psychiatry Investig 6(4):245-254, https://doi.org/10.4306/pi.2009.6.4.245.

Klemm W, Heusinkveld BG, Lenzholzer S, Jacobs MH, Van Hove B. 2015. Psychological and physical impact of urban green spaces on outdoor thermal comfort during summertime in the Netherlands. Build Environ 83:120-128, https://doi.org/10.1016/j.buildenv.2014.05.013.

Kobayashi H, Song C, Ikei H, Kagawa T, Miyazaki Y. 2015. Analysis of individual variations in autonomic responses to urban and forest environments. Evid Based Complement Alternat Med 2015:61094, https:doi.org/http://dx.doi.org/10.1155/2015/671094.

Kondo MC, Han S, Donovan GH, MacDonald JM. 2017a. The association between urban trees and crime: Evidence from the spread of the emerald ash borer in Cincinnati. Landsc Urban Plan 157:193-199, https://doi.org/10.1016/j.landurbplan.2016.07.003.

Kondo MC, South EC, Branas CC, Richmond TS, Wiebe DJ. 2017b. The association between urban tree cover and gun assault: A case-control and case-crossover study. Am J Epidemiol 186(3):289-296, https://doi.org/10.1093/aje/kwx096.

Kühn S, Düzel S, Eibich P, Krekel C, Wüstemann H, Kolbe J, Martensson J, Goebel J, Gallinat J, Wagner GG. 2017. In search of features that constitute an "enriched environment" in humans: Associations between geographical properties and brain structure. Sci Rep 7:11920, https://doi.org/10.1038/s41598-017-12046-7.

Kuo FE 2001. Coping with poverty: Impacts of environment and attention in the inner city. Environ Behav 33(1):5-34, https://doi.org/10.1177/00139160121972846.

Kuo FE, Sullivan WC. 2001a. Aggression and violence in the inner city: Effects of environment via mental fatigue. Environ Behav 33(4):543-571, https://doi.org/10.1177/00139160121973124.

Kuo FE, Sullivan WC. 2001b. Environment and crime in the inner city: Does vegetation reduce crime? Environ Behav 33(3):343-367, https://doi.org/10.1177/0013916501333002.

Larsen K, Gilliland J, Hess P, Tucker P, Irwin J, He M. 2009. The influence of the physical environment and sociodemographic characteristics on children's mode of travel to and from school. Am J Public Health 99(3):520-526, https://doi.org/10.2105/AJPH.2008.135319.

Lee H, Mayer H, Chen L. 2016. Contribution of trees and grasslands to the mitigation of human heat stress in a residential district of Freiburg, southwest Germany. Landsc Urban Plan 148:37-50, https://doi.org/10.1016/j.landurbplan.2015.12.004.

Lee J, Park BJ, Tsunetsugu Y, Kagawa T, Miyazaki Y. 2009. Restorative effects of viewing real forest landscapes, based on a comparison with urban landscapes. Scand J For Res 24(3):227-234, https://doi.org/10.1080/02827580902903341.

Lee J, Park BJ, Tsunetsugu Y, Ohira T, Kagawa T, Miyazaki Y. 2011. Effect of forest bathing on physiological and psychological responses in young Japanese male subjects. Public Health 125(2):93-100, https://doi.org/10.1016/j.puhe.2010.09.005.

Lee J, Tsunetsugu Y, Takayama N, Park B-J, Li Q, Song C, Komatsu M, Ikei H, Tyrväinen L, Kagawa T. 2014. Influence of forest therapy on cardiovascular relaxation in young adults. J Evid Based Complementary Altern Med 2014:834360, http://dx.doi.org/10.1155/2014/834360.

Li J, Sun B, Huang Y, Lin X, Zhao D, Tan G, Wu J, Zhao H, Cao L, et al. 2009. A multicentre study assessing the prevalence of sensitizations in patients with asthma and/or rhinitis in China. Allergy 64(7):1083-1092, https://doi.org/10.1111/j.1398-9995.2009.01967.x.

Li Q, Kobayashi M, Inagaki H, Hirata Y, Li YJ, Hirata K, Shimizu T, Suzuki H, Katsumata M, et al. 2010. A day trip to a forest park increases human natural killer activity and the expression of anti-cancer proteins in male subjects. J Biol Regul Homeost Agents 24(2):157-166.

Li Q, Kobayashi M, Kawada T. 2008a. Relationships between percentage of forest coverage and standardized mortality ratios (SMR) of cancers in all prefectures in Japan. Open Public Health J 1:1-7.

Li Q, Morimoto K, Kobayashi M, Inagaki H, Katsumata M, Hirata Y, Hirata K, Shimizu T, Li YJ, et al. 2008b. A forest bathing trip increases human natural killer activity and expression of anti-cancer proteins in female subjects. J Biol Regul Homeost Agents 22(1):45-55.

Li Q, Morimoto K, Kobayashi M, Inagaki H, Katsumata M, Hirata Y, Hirata K, Suzuki H, Li YJ, et al. 2008c. Visiting a forest, but not a city, increases human natural killer activity and expression of anti-cancer proteins. Int J Immunopathol Pharmacol 21(1):117-127, https://doi.org/10.1177%2F039463200802100113.

Li Q, Morimoto K, Nakadai A, Inagaki H, Katsumata M, Shimizu T, Hirata Y, Hirata K, Suzuki H, et al. 2007. Forest bathing enhances human natural killer activity and expression of anti-cancer proteins. Int J Immunopathol Pharmacol 20(2 Suppl 2):3-8, https://doi.org/10.1177%2F03946320070200S202.

Li Q, Otsuka T, Kobayashi M, Wakayama Y, Inagaki H, Katsumata M, Hirata Y, Li YJ, Hirata K, et al. 2011. Acute effects of walking in forest environments on cardiovascular and metabolic parameters. Eur J Appl Physiol 111(11):2845-2853, https://doi.org/10.1007/s00421-011-1918-z.

Lin RY, Clauss AE, Bennett ES. 2002. Hypersensitivity to common tree pollens in New York City patients. Allergy Asthma Proc 23(4):253-8.

Lin T, Matzarakis A, Hwang R. 2010. Shading effect on long-term outdoor thermal comfort. Build Environ 45(1):213-221, https://doi.org/10.1016/j.buildenv.2009.06.002.

Lin T, Tsai K, Hwang R, Matzarakis A. 2012. Quantification of the effect of thermal indices and sky view factor on park attendance. Landsc Urban Plan 107(2):137-146, https://doi.org/10.1016/j.landurbplan.2012.05.011.

Lin T, Tsai K, Liao C, Huang Y. 2013. Effects of thermal comfort and adaptation on park attendance regarding different shading levels and activity types. Build Environ 59:599-611, https://doi.org/10.1016/j.buildenv.2012.10.005.

Lin YH, Tsai CC, Sullivan WC, Chang PJ, Chang CY. 2014. Does awareness effect the restorative function and perception of street trees? Front Psychol 5:906, https://doi.org/10.3389/fpsyg.2014.00906.

Loureiro G, Rabaca M-, Blanco B, Andrade S, Chieira C, Pereira C. 2005. Urban versus rural environment--any differences in aeroallergens sensitization in an allergic population of Cova da Beira, Portugal? Eur Ann Allergy Clin Immunol 37(5):187-193.

Lovasi GS, Bader MD, Quinn J, Neckerman K, Weiss C, Rundle A. 2012. Body mass index, safety hazards, and neighbourhood attractiveness. Am J Prev Med 43(4):378-384, https://doi.org/10.1016/j.amepre.2012.06.018.

Lovasi GS, Jacobson JS, Quinn JW, Neckerman KM, Ashby-Thompson MN, Rundle A. 2011. Is the environment near home and school associated with physical activity and adiposity of urban preschool children? J Urban Health 88(6):1143-1157, https://doi.org/10.1007/s11524-011-9604-3.

Lovasi GS, O'Neil-Dunne JP, Lu JW, Sheehan D, Perzanowski MS, MacFaden SW, King KL, Matte T, Miller RL, Hoepner LA. 2013a. Urban tree canopy and asthma, wheeze, rhinitis, and allergic sensitization to tree pollen in a New York City birth cohort. Environ Health Perspect 121(4):494-500, https://doi.org/10.1289/ehp.1205513.

Lovasi GS, Quinn JW, Neckerman KM, Perzanowski MS, Rundle A. 2008. Children living in areas with more street trees have lower prevalence of asthma. J Epidemiol Community Health 62:647-649, https://doi.org/10.1136/jech.2007.071894.

Lovasi GS, Schwartz-Soicher O, Neckerman KM, Konty K, Kerker B, Quinn J, Rundle A. 2013b. Aesthetic amenities and safety hazards associated with walking and bicycling for transportation in New York City. Ann Behav Med 45 Suppl 1:S76-S85, https://doi.org/10.1007/s12160-012-9416-z.

Lovasi GS, Schwartz-Soicher O, Quinn JW, Berger DK, Neckerman KM, Jaslow R, Lee KK, Rundle A. 2013c. Neighbourhood safety and green space as predictors of obesity among preschool children from low-income families in New York City. Prev Med 57(3):189-193, https://doi.org/10.1016/j.ypmed.2013.05.012.

Mao G-X, Cao Y-B, Lan X-G, He Z-H, Chen Z-M, Wang Y-Z, Hu X-L, Lv Y-D, Wang G-F, Yan J. 2012a. Therapeutic effect of forest bathing on human hypertension in the elderly. J Cardiol 60(6):495-502, https://doi.org/10.1016/j.jjcc.2012.08.003.

Mao GX, Lan XG, Cao YB, Chen ZM, He ZH, Lv YD, Wang YZ, Hu XL, Wang GF, Yan J. 2012b. Effects of short-term forest bathing on human health in a broad-leaved evergreen forest in Zhejiang Province, China. Biomed Environ Sci 25(3):317-324, https://doi.org/10.3967/0895-3988.2012.03.010.

Markevych I, Smith MP, Jochner S, Standl M, Brüske I, von Berg A, Bauer CP, Fuks K, Koletzko S, et al. 2016. Neighbourhood and physical activity in German adolescents: GINIplus and LISAplus. Environ Res 147:284-293, https://doi.org/10.1016/j.envres.2016.02.023.

Martens D, Gutscher H, Bauer N. 2011. Walking in "wild" and "tended" urban forests: The impact on psychological well-being. J Environ Psychol 31(1):36-44, https://doi.org/10.1016/j.jenvp.2010.11.001.

Mårtensson F, Boldemann C, Söderström M, Blennow M, Englund JE, Grahn P. 2009. Outdoor environmental assessment of attention promoting settings for preschool children. Health Place 15(4):1149-57, https://doi.org/10.1016/j.healthplace.2008.11.001.

Martínez-Soto J, Gonzales-Santos L, Pasaye E, Barrios FA. 2013. Exploration of neural correlates of restorative environment exposure through functional magnetic resonance. Intelligent Buildings International 5(sup1):10-28, https://doi.org/10.1080/17508975.2013.807765.

Matsunaga K, Park B-J, Kobayashi H, Miyazaki Y. 2011. Physiologically relaxing effect of a hospital rooftop forest on older women requiring care. J Am Geriatr Soc 59(11):2162-2163, https://doi.org/10.1111/j.1532-5415.2011.03651.x.

May L, Carim M, Yadav K. 2011. Adult asthma exacerbations and environmental triggers: A retrospective review of ED visits using an electronic medical record. Am J Emerg Med 29(9):1074-1082, https://doi.org/10.1016/j.ajem.2010.06.034.

Mertens L, Compernolle S, Deforche B, Mackenbach JD, Lakerveld J, Brug J, Roda C, Feuillet T, Oppert JM, et al. 2017. Built environmental correlates of cycling for transport across Europe. Health Place 44:35-42, https://doi.org/10.1016/j.healthplace.2017.01.007.

Milošević DD, Bajšanski IV, Savić SM. 2017. Influence of changing trees locations on thermal comfort on street parking lot and footways. Urban For Urban Gree 23:113-124, https://doi.org/10.1016/j.ufug.2017.03.011.

Morita E, Fukuda S, Nagano J, Hamajima N, Yamamoto H, Iwai Y, Nakashima T, Ohira H, Shirakawa T. 2007. Psychological effects of forest environments on healthy adults: Shinrin-yoku (forest-air bathing, walking) as a possible method of stress reduction. Public Health 121(1):54-63, https://doi.org/10.1016/j.puhe.2006.05.024.

Motreff Y, Golliot F, Calleja M, Le Pape A, Fuhrman C, Farrera I, Plaisant I. 2014. Short-term effect of pollen exposure on drug consumption for allergic rhinitis and conjunctivitis. Aerobiologia 30(1):35-44, https://doi.org/10.1007/s10453-013-9307-1.

Nehme EK, Oluyomi AO, Calise TV, Kohl HW. 2016. Environmental correlates of recreational walking in the neighbourhood. Am J Health Promot 30(3):139-148, https://doi.org/10.4278/ajhp.130531-QUAN-281.

Nordh H, Grahn P, Währborg P. 2009. Meaningful activities in the forest, a way back from exhaustion and long-term sick leave. Urban For Urban Gree 8(3):207-219, https://doi.org/10.1016/j.ufug.2009.02.005.

Nowak DJ, Hirabayashi S, Bodine A, Greenfield E. 2014. Tree and forest effects on air quality and human health in the United States. Environ Pollut 193:119-129, https://doi.org/10.1016/j.envpol.2014.05.028.

Nowak DJ, Hirabayashi S, Bodine A, Hoehn R. 2013. Modeled PM2.5 removal by trees in ten U.S. cities and associated health effects. Environ Pollut 178:395-402, https://doi.org/10.1016/j.envpol.2013.03.050.

Ohtsuka Y, Yabunaka N, Takayama S. 1998. Shinrin-yoku (forest-air bathing and walking) effectively decreases blood glucose levels in diabetic patients. Int J Biometeorol 41(3):125-127, https://doi.org/10.1007/s004840050064.

Osborne NJ, Alcock I, Wheeler BW, Hajat S, Sarran C, Clewlow Y, McInnes RN, Hemming D, White M, Vardoulakis S, Fleming LE. 2017. Pollen exposure and hospitalization due to asthma exacerbations: Daily time series in a European city. Int J Biometeorol 61(10):1837-1848, https://doi.org/10.1007/s00484-017-1369-2.

Paddle E, Gilliland J. 2016. Orange is the new green: Exploring the restorative capacity of seasonal foliage in schoolyard trees. Int J Environ Res Public Health 13(5):e18, https://doi.org/10.3390/ijerph13050497.

Parisi AV, Kimlin MG, Wong JCF, Lester R, Turnbull D. 2000a. Reduction in the personal annual solar erythemal ultraviolet exposure provided by Australian gum trees. Radiat Prot Dosimetry 92(4):307-312, https://doi.org/10.1093/oxfordjournals.rpd.a033297.

Parisi AV, Kimlin MG, Wong JCF, Wilson M. 2000b. Personal exposure distribution of solar erythemal ultraviolet radiation in tree shade over summer. Phys Med Biol 45(2):349, http://doi.org/10.1088/0031-9155/45/2/307.

Park B-J, Furuya K, Kasetani T, Takayama N, Kagawa T, Miyazaki Y. 2011. Relationship between psychological responses and physical environments in forest settings. Landsc Urban Plan 102(1):24-32, https://doi.org/10.1016/j.landurbplan.2011.03.005.

Park B-J, Tsunetsugu Y, Kasetani T, Hirano H, Kagawa T, Sato M, Miyazaki Y. 2007. Physiological effects of shinrin-yoku (taking in the atmosphere of the forest)-using salivary cortisol and cerebral activity as indicators. J Physiol Anthropol 26(2):123-128, https://doi.org/10.2114/jpa2.26.123.

Park B-J, Tsunetsugu Y, Kasetani T, Kagawa T, Miyazaki Y. 2010. The physiological effects of shinrin-yoku (taking in the forest atmosphere or forest bathing): Evidence from field experiments in 24 forests across Japan. Environ Health Prev Med 15(1):18-26, https://doi.org/10.1007/s12199-009-0086-9.

Park B-J, Tsunetsugu Y, Kasetani T, Morikawa T, Kagawa T, Miyazaki Y. 2009. Physiological effects of forest recreation in a young conifer forest in Hinokage Town, Japan. Silva Fennica 43(2):291-301.

Perkins S, Searight HR, Ratwik S. 2011. Walking in a natural winter setting to relieve attention fatigue: A pilot study. Psychology 2(8):777-780, https://doi.org/10.4236/psych.2011.28119.

Piff PK, Dietze P, Feinberg M, Stancato DM, Keltner D. 2015. Awe, the small self, and prosocial behavior. J Pers Soc Psychol 108(6):883-899, https://doi.org/10.1037/pspi0000018.

Pilat MA, McFarland A, Snelgrove A, Collins K, Waliczek TM, Zajicek J. 2012. The effect of tree cover and vegetation on incidence of childhood asthma in Metropolitan Statistical Areas of Texas. HortTechnology 22(5):631-637, https://doi.org/10.21273/HORTTECH.22.5.631.

Puiggròs A, Muñoz-Cano R, Roger Reig A, Raga E, Belmonte J, Valero A. 2015. Prevalence of sensitization to pollen from trees planted in Barcelona City. J Investig Allergol Clin Immunol 25(2):150-1.

Rao M, George LA, Rosenstiel TN, Shandas V, Dinno A. 2014. Assessing the relationship among urban trees, nitrogen dioxide, and respiratory health. Environ Pollut 194:96-104, https://doi.org/10.1016/j.envpol.2014.07.011.

Rao M, George LA, Shandas V, Rosenstiel TN. 2017. Assessing the potential of land use modification to mitigate ambient NO_2 and its consequences for respiratory health. Int J Environ Res Public Health 14(7), https://doi.org/10.3390/ijerph14070750.

Ratola N, Jiménez-Guerrero P. 2017. Modelling benzo[a]pyrene in air and vegetation for different land uses and assessment of increased health risk in the Iberian Peninsula. Environ Sci Pollut Res Int 24(13):11901-11910, https://doi.org/10.1007/s11356-015-5394-6.Ren Y, Qu Z, Du Y, Xu R, Ma D,

Reid CE, Clougherty JE, Shmool JLC, Kubzansky LD. 2017. Is all urban green space the same? A comparison of the health benefits of trees and grass in New York City. Int J Environ Res Public Health 14(11) :1411; https://doi.org/10.3390/ijerph14111411.

Ren Y, Qu Z, Du Y, Xu R, Ma D, Yang G, Shi Y, Fan X, Tani A, et al. 2017. Air quality and health effects of biogenic volatile organic compounds emissions from urban green spaces and the mitigation strategies. Environ Pollut 230:849-861, https://doi.org/10.1016/j.envpol.2017.06.049.

Roe J, Aspinall P. 2011. The restorative outcomes of forest school and conventional school in young people with good and poor behaviour. Urban For Urban Gree 10:205-212. https://doi.org/10.1016/j.ufug.2011.03.003.

Sam CK, Soon SC, Liam CK, Padmaja K, Cheng HM. 1998. An investigation of aeroallergens affecting urban Malaysian asthmatics. Asian Pac J Allergy Immunol 16(1):17-20.

Sánchez-Mesa JA, Serrano P, Cariñanos P, Prieto-Baena JC, Moreno C, Guerra F, Galan C. 2005. Pollen allergy in Cordoba city: Frequency of sensitization and relation with antihistamine sales. J Investig Allergol Clin Immunol 15(1):50-56.

Sanusi R, Johnstone D, May P, Livesley SJ. 2016. Street orientation and side of the street greatly influence the microclimatic benefits street trees can provide in summer. J Environ Qual 45(1):167-174, https://doi.org/ 10.2134/jeq2015.01.0039.

Schipperijn J, Bentsen P, Troelsen J, Toftager M, Stigsdotter UK. 2013. Associations between physical activity and characteristics of urban green space. Urban For Urban Gree 12(1):109-116, https://doi.org/10.1016/j.ufug.2012.12.002.

Seo SC, Park SJ, Park C-W, Yoon WS, Choung JT, Yoo Y. 2014. Clinical and immunological effects of a forest trip in children with asthma and atopic dermatitis. Iran J Allergy Asthma Immunol 14(1):28-36.

Sheehan WJ, Rangsithienchai PA, Baxi SN, Gardynski A, Bharmanee A, Israel E, Phipatanakul W. 2010. Age-specific prevalence of outdoor and indoor aeroallergen sensitization in Boston. Clin Pediatr (Phila) 49(6):579-585, https:doi.org/10.1177/0009922809354326.

Sheffield PE, Weinberger KR, Ito K, Matte TD, Mathes RW, Robinson GS, Kinney PL. 2011. The association of tree pollen concentration peaks and allergy medication sales in New York City: 2003-2008. ISRN Allergy 2011:537194.

Shin WS. 2007. The influence of forest view through a window on job satisfaction and job stress. Scand J For Res 22(3):248-253, https://doi.org/10.1080/02827580701262733.

Shin WS, Shin CS, Yeoun PS, Kim JJ. 2011. The influence of interaction with forest on cognitive function. Scand J For Res 26(6):595-598, https://doi.org/10.1080/02827581.2011.585996.

Shin Y-K, Kim DJ, Jung-Choi K, Son Y-J, Koo J-W, Min J-A, Chae J-H. 2013. Differences of psychological effects between meditative and athletic walking in a forest and gymnasium. Scand J For Res 28(1):64-72, https://doi.org/10.1080/02827581.2012.706634.

Song C, Ikei H, Kobayashi M, Miura T, Li Q, Kagawa T, Kumeda S, Imai M, Miyazaki Y. 2017a. Effects of viewing forest landscape on middle-aged hypertensive men. Urban For Urban Gree 21:247-252, https://doi.org/10.1016/j.ufug.2016.12.010.

Song C, Ikei H, Kobayashi M, Miura T, Taue M, Kagawa T, Li Q, Kumeda S, Imai M, Miyazaki Y. 2015a. Effect of forest walking on autonomic nervous system activity in middle-aged hypertensive individuals: A pilot study. Int J Environ Res Public Health 12(3):2687-2699.

Song C, Ikei H, Lee J, Park B-J, Kagawa T, Miyazaki Y. 2013. Individual differences in the physiological effects of forest therapy based on type A and type B behavior patterns. J Physiol Anthropol 32:14.

Song C, Ikei H, Miyazaki Y. 2015. Elucidation of a physiological adjustment effect in a forest environment: A pilot study. Int J Environ Res Public Health 12(4):4247-4255, https://doi.org/10.3390/ijerph120404247.

Song C, Ikei H, Miyazaki Y. 2017b. Sustained effects of a forest therapy program on the blood pressure of office workers. Urban For Urban Gree 27:246-252, https://doi.org/10.1016/j.ufug.2017.08.015.

Song GS, Jeong MA. 2016. Morphology of pedestrian roads and thermal responses during summer, in the urban area of Bucheon City, Korea. Int J Biometeorol 60(7):999-1014, https://doi.org/10.1007/s00484-015-1092-9.

Song X, Li H, Li C, Xu J, Hu D. 2016. Effects of VOCs from leaves of *Acer truncatum Bunge* and *Cedrus deodara* on human physiology and psychology. Urban For Urban Gree 19:29-34, https://doi.org/10.1016/j.ufug.2016.06.021.

Sonntag-Öström E, Nordin M, Lundell Y, Dolling A, Wiklund U, Karlsson M, Carlberg B, Slunga Järvholm L. 2014. Restorative effects of visits to urban and forest environments in patients with exhaustion disorder. Urban For Urban Gree 13(2):344-354, https://doi.org/10.1016/j.ufug.2013.12.007.

Stone B, Vargo J, Liu P, Habeeb D, DeLucia A, Trail M, Hu Y, Russell A. 2014. Avoided heat-related mortality through climate adaptation strategies in three US cities. PLoS One 9(6):e100852, https://doi.org/10.1371/journal.pone.0100852.

Sullivan WC, Kuo FE, DePooter S. 2004. The fruit of urban nature: Vital neigborhood spaces. Environ Behav 36(5):678-700, https://doi.org/10.1177/0193841X04264945.

Sung M, Kim SW, Kim JH, Lim DH. 2017. Regional difference of causative pollen in children with allergic rhinitis. J Korean Med Sci 32(6):926-932, https:doi.org/10.3346/jkms.2017.32.6.926.

Sung J, Woo JM, Kim W, Lim SK, Chung EJ. 2012. The effect of cognitive behavior therapy-based “'forest therapy”' program on blood pressure, salivary cortisol level, and quality of life in elderly hypertensive patients. Clin Exp Hypertens 34(1):1-7, https://doi.org/10.3109/10641963.2011.618195.

Takano T, Nakamura K, Watanabe M. 2002. Urban residential environments and senior citizens’ longevity in megacity areas: The importance of walkable green spaces. J Epidemiol Community Health 56(12):913-916, https://doi.org/10.1136/jech.56.12.913.

Takayama N, Korpela K, Lee J, Morikawa T, Tsunetsugu Y, Park B-J, Li Q, Tyrväinen L, Miyazaki Y, Kagawa T. 2014. Emotional, restorative and vitalizing effects of forest and urban environments at four sites in Japan. Int J Environ Res Public Health 11(7):7207-7230.

Tarar G, Etheredge CL, McFarland A, Snelgrove A, Waliczek TM, Jajicek JM. 2015. The effect of urban tree canopy cover and vegetation levels on incidence of stress-related illnesses in humans in Metropolitan Statistical Areas of Texas. HortTechnology 25(1):76-84, https://doi.org/10.21273/HORTTECH.25.1.76.

Taylor AF, Kuo FE, Sullivan WC. 2002. Views of nature and self-discipline: Evidence from inner city children. J Environ Psychol 22(1-2):49-63, https://doi.org/10.1006/jevp.2001.0241.

Taylor AF, Wiley A, Kuo FE, Sullivan WC. 1998. Growing up in the inner city: Green spaces as places to grow. Environ Behav 30(1):3-27, https://doi.org/10.1177/0013916598301001.

Taylor MS, Wheeler BW, White MP, Economou T, Osborne NJ. 2015. Urban street tree density and antidepressant prescription rates—A cross-sectional study in London, UK. Landsc Urban Plan 136:174-179, https://doi.org/10.1016/j.landurbplan.2014.12.005.

Tilt JH. 2010. Walking trips to parks: Exploring demographic, environmental factors, and preferences for adults with children in the household. Prev Med 50 Suppl 1:S69-S73, https://doi.org/10.1016/j.ypmed.2009.07.026.

Tiwary A, Sinnett D, Peachey C, Chalabi Z, Vardoulakis S, Fletcher T, Leonardi G, Grundy C, Azapagic A, Hutchings TR. 2009. An integrated tool to assess the role of new planting in PM10 capture and the human health benefits: A case study in London. Environ Pollut 157(10):2645-2653, https://doi.org/10.1016/j.envpol.2009.05.005.

Toda M, Den R, Hasegawa-Ohira M, Morimoto K. 2013. Effects of woodland walking on salivary stress markers cortisol and chromogranin A. Complement Ther Med 21(1):29-34, https://doi.org/10.1016/j.ctim.2012.11.004.

Townsend JB, Ilvento TW, Barton SS. 2016. Exploring the relationship between trees and human stress in the urban environment. Arb UF 42(3):146-159.

Troy A, Grove MJ, O'Neil-Dunne J. 2012. The relationship between tree canopy and crime rates across an urban--rural gradient in the greater Baltimore region. Landsc Urban Plan 106(3):262-270, https://doi.org/10.1016/j.landurbplan.2012.03.010.

Tsai WL, Floyd MF, Leung YF, McHale MR, Reich BJ. 2016. Urban vegetative cover fragmentation in the U.S.: Associations with physical activity and BMI. Am J Prev Med 50(4):509-517, https://doi.org/10.1016/j.amepre.2015.09.022.

Tsunetsugu Y, Lee J, Park B-J, Tyrväinen L, Kagawa T, Miyazaki Y. 2013. Physiological and psychological effects of viewing urban forest landscapes assessed by multiple measurements. Landsc Urban Plan 113:90-93, https://doi.org/10.1016/j.landurbplan.2013.01.014.

Tsunetsugu Y, Park BJ, Ishii H, Hirano H, Kagawa T, Miyazaki Y. 2007. Physiological effects of shinrin-yoku (taking in the atmosphere of the forest) in an old-growth broadleaf forest in Yamagata Prefecture, Japan. J Physiol Anthropol 26(2):135-142, https://doi.org/10.2114/jpa2.26.135.

Tyrväinen L, Ojala A, Korpela K, Lanki T, Tsunetsugu Y, Kagawa T. 2014. The influence of urban green environments on stress relief measures: A field experiment. J Environ Psychol 38:1 - 9, https://doi.org/10.1016/j.jenvp.2013.12.005.

Ulmer JM, Wolf KL, Backman DR, Tretheway RL, Blain CJ, ONeil-Dunne JP, Frank LD. 2016. Multiple health benefits of urban tree canopy: The mounting evidence for a green prescription. Health Place 42:54-62, https://doi.org/10.1016/j.healthplace.2016.08.011.

Ulrich RS. 1984. View through a window may influence recovery from surgery. Science 224(4647):420-421, https://doi.org/10.1126/science.6143402.

Van den Berg AE, Jorgensen A, Wilson ER. 2014. Evaluating restoration in urban green spaces: Does setting type make a difference? Landscape and Urban Planning 127:173-181, https://doi.org/10.1016/j.landurbplan.2014.04.012.

Van den Berg AE, Van den Berg CG. 2011. A comparison of children with ADHD in a natural and built setting. Child Care Health Dev 37(3):430-439, https://doi.org/10.1111/j.1365-2214.2010.01172.x.

Vieira FA, Ferreira EN, Cruz AA. 1998. Grass allergy increases the risk of tree pollen sensitization: a warning to urban planners. J Allergy Clin Immunol 102(4 Pt 1):700-1, https://doi.org/10.1016/S0091-6749(98)70293-3.

Wang L, Zhao X, Xu W, Tang J, Jiang X. 2016. Correlation analysis of lung cancer and urban spatial factor: Based on survey in Shanghai. J Thorac Dis 8(9):2626-2637, https://doi.org/10.21037/jtd.2016.09.10.

Weichenthal S, Lavigne E, Villeneuve PJ, Reeves F. 2016. Airborne pollen concentrations and emergency room visits for myocardial infarction: A multicity case-crossover study in Ontario, Canada. Am J Epidemiol 183(7):613-621, https://doi.org/10.1093/aje/kwv252.

Wilson LA, Giles-Corti B, Burton NW, Giskes K, Haynes M, Turrell G. 2011. The association between objectively measured neighbourhood features and walking in middle-aged adults. Am J Health Promot 25(4):e12-21, https://doi.org/10.1080/13549839.2011.646965.

Wolf LJ, Zu Ermgassen S, Balmford A, White M, Weinstein N. 2017. Is variety the spice of life? An experimental investigation into the effects of species richness on self-reported mental well-being. PLoS One 12(1):e0170225, https://doi.org/10.1371/journal.pone.0170225.

Wu J, Jackson L. 2017. Inverse relationship between urban green space and childhood autism in California elementary school districts. Environ Int 107:140-146, https://doi.org/10.1016/j.envint.2017.07.010.

Wu J, Rappazzo KM, Simpson RJ, Joodi G, Pursell IW, Mounsey JP, Cascio WE, Jackson LE. 2018. Exploring links between greenspace and sudden unexpected death: A spatial analysis. Environ Int 113:114-121, https://doi.org/10.1016/j.envint.2018.01.021.

Wu Z, Kong F, Wang Y, Sun R, Chen L. 2016. The impact of greenspace on thermal comfort in a residential quarter of Beijing, China. Int J Environ Res Public Health 13(1217), https//:doi.org/10.3390/ijerph13121217.

Yamaguchi M, Deguchi M, Miyazaki Y. 2006. The effects of exercise in forest and urban environments on sympathetic nervous activity of normal young adults. J Int Med Res 34(2):152-159, https://doi.org/10.1177/147323000603400204.

Yu CP, Lin CM, Tsai MJ, Tsai YC, Chen CY. 2017. Effects of short forest bathing program on autonomic nervous system activity and mood states in middle-aged and elderly individuals. Int J Environ Res Public Health 14:897, https://doi.org/10.3390/ijerph14080897.

Zeng Y, Dong L. 2015. Thermal human biometeorological conditions and subjective thermal sensation in pedestrian streets in Chengdu, China. Int J Biometeorol 59(1):99-108, https://doi.org/10.1007/s00484-014-0883-8.

Zuniga-Teran AA, Orr BJ, Gimblett RH, Chalfoun NV, Guertin DP, Marsh SE. 2017. Neighbourhood design, physical activity, and wellbeing: Applying the walkability model. Int J Environ Res Public Health 14(1):76, https://doi.org/10.3390/ijerph14010076.
